# Identification of the canonical and noncanonical role of miR-143/145 in estrogen-deficient bone loss

**DOI:** 10.7150/thno.55041

**Published:** 2021-03-13

**Authors:** Rongyao Xu, Xin Shen, Hanyu Xie, Hengguo Zhang, Dingshan Liu, Xin Chen, Yu Fu, Ping Zhang, Yi Yang, Jie Cheng, Hongbing Jiang

**Affiliations:** 1 Jiangsu Key Laboratory of Oral Diseases, Nanjing Medical University, Nanjing 210029, Jiangsu Province, China.; 2 Department of Oral and Maxillofacial Surgery, Affiliated Hospital of Stomatology, Nanjing Medical University, Nanjing 210029, Jiangsu Province, China.; 3 Department of Dental Implantology, Affiliated Hospital of Stomatology, Nanjing Medical University, Nanjing 210029, Jiangsu Province, China.

**Keywords:** Estrogen, Bone Mesenchymal Stem Cells (BMSCs), core transcription factors (TFs), miR-143/145, Extracellular Vesicles (EVs).

## Abstract

**Rationale:** Postmenopausal-induced bone loss is mainly caused by declining core transcription factors (TFs) of bone mesenchymal stem cells (BMSCs), but little is known about how miRNAs regulate chromatin structure remodeling of TFs gene to maintain BMSCs function in bone homeostasis.

**Methods:** We examined the serum, salivary and bone samples from Pre- and Post-menopause women by paired analysis and confirmed canonical ceRNA role of MIR143HG and miR-143/145 complexes in cytoplasm and noncanonical role for SOX2 transcription in nucleus (FISH, qRT-PCR, immunostaining, Luciferase assays and ChIP). Moreover, we took advantage of transgenic mice under OVX-induced osteoporosis, studying the *in vitro* and *in vivo* effect of miR-143/145 deletion on BMSCs function and bone homeostasis. Last, using miRNA antagonism, antagomiR-143/145 were delivered into bone marrow to treat estrogen-deficient bone loss.

**Results:** Here, we identified miR-143/145 as potential diagnostic candidates for postmenopausal osteoporosis, and miR-143/145 overexpression impaired BMSCs self-renewing and differentiation function. Mechanistically, we confirmed that cytoplasmic miR-143/145 and LncRNA MIR143HG, that controlled by ERβ, cooperatively regulated pluripotency genes translation via canonical ceRNA pathway, and MIR143HG cooperates with miR‑143 to nuclear translocation for co-activation of SOX2 transcription via opening promoter chromatin. Meanwhile, miR‑143/145 were shuttled into osteoclasts in extracellular vesicles and triggered osteoclastic activity by targeting Cd226 and Srgap2. Furthermore, *miR-143/145^-/-^*mice or using chemically‑modified antagomiR-143/145 significantly alleviated estrogen-deficient osteoporosis.

**Conclusions:** Our findings reveal a canonical and noncanonical role of miR-143/145 in controlling BMSCs pluripotency and unfold their dual effect on bone formation and bone resorption, suggesting miR-143/145 as promising therapeutic targets for treating estrogen-deficient bone loss.

## Introduction

In postmenopausal women, estrogen deficiency leads to imbalanced bone homeostasis that is characterized by increased bone destruction and decreased bone rebuilding, which is mainly caused by defective self-renewing and aberrant differentiation of bone mesenchymal stem cells (BMSCs) 1-3. The core transcription factors (TFs) NANOG, SOX2, OCT4 act as central regulators to maintain stem cell self-renewing and multipotency 4, 5. Loss of core TFs results in genomic instability and irreversible senescence state of somatic cells due to progressive accumulation of epigenetic errors in chromatin 6, 7, as well as failure initiation of osteogenic signaling of MSCs 8. These core TFs expression are controlled by many extrinsic signaling, including WNT/beta-catenin signaling, FGF signaling and hormone signaling 9, 10. Estrogen receptors, as activated by estrogen, are widespread expressed on BMSCs 11 and especially, estrogen receptor β (ERβ) plays a crucial role in regulating TFs network and maintain self-renewing and multipotency 12. Thus, understanding how estrogen regulates these core TFs of BMSCs during postmenopausal bone loss is crucial for the development of new osteoporosis treatment strategies.

Multiple miRNAs have been identified that post-transcriptionally control BMSCs self-renewal and multipotency, by combining with Argonaute (AGO) proteins to target mRNA in RNA induced silencing complexes (RISCs) 13-15. Here, we screened the expression profiles of BMSCs from premenopausal or postmenopausal women and identified two miRNAs, miR‑143 and miR‑145. As a miRNA cluster, miR-143/145 may share some cis acting elements and cooperate for efficient functional regulation 16-18. Recent evidence suggests that miR-143 and lncRNA MIR143HG cooperated core TFs posttranscriptional activity through canonical competing endogenous RNAs (ceRNAs) mechanism 19. Although served as a miRNA sponge, it is still unknown whether the MIR143HG and miR-143 complexes are existed as a set of quiescent silencers in cytoplasm or functional modulators to exert following biological activity. Here, we first uncovered that upon the formation of the complexes, they will localize to the nuclei and pair with the promoter of SOX2 to opening local chromatin. Our results extend the current understanding of ceRNAs biological action that control gene expression in both transcription and post-transcription.

In this study, we aimed to determine whether and by what mechanisms miRNAs regulate core TFs in cytoplasm and nuclei. We identified miR-143/145 cluster as key governors in the maintenance of bone homeostasis and whose function were coupled with MIR143HG under the direct modulation of pluripotent gene in transcription and post-transcription. Moreover, we showed that BMSCs-derived miR-143/145 regulated osteoclast differentiation via shedding extracellular vesicles (EVs) to transfer miR-143/145 into osteoclasts. To further explore the therapeutic potential of miR-143/145, we generated ovariectomized (OVX) wild type (WT) and miR-143/145 knock out mice, and confirmed that loss of miR-143/145 could prevent bone loss and promote bone regeneration in estrogen-deficient mice. Therefore, miR-143/145 may be potential target for the development of diagnostic and therapeutic treatments for osteoporosis.

## Results

### Increases in miR-143/145 levels during estrogen-deficient osteoporosis and the attenuation of self-renewing and osteogenic differentiation

**Figure 1:**
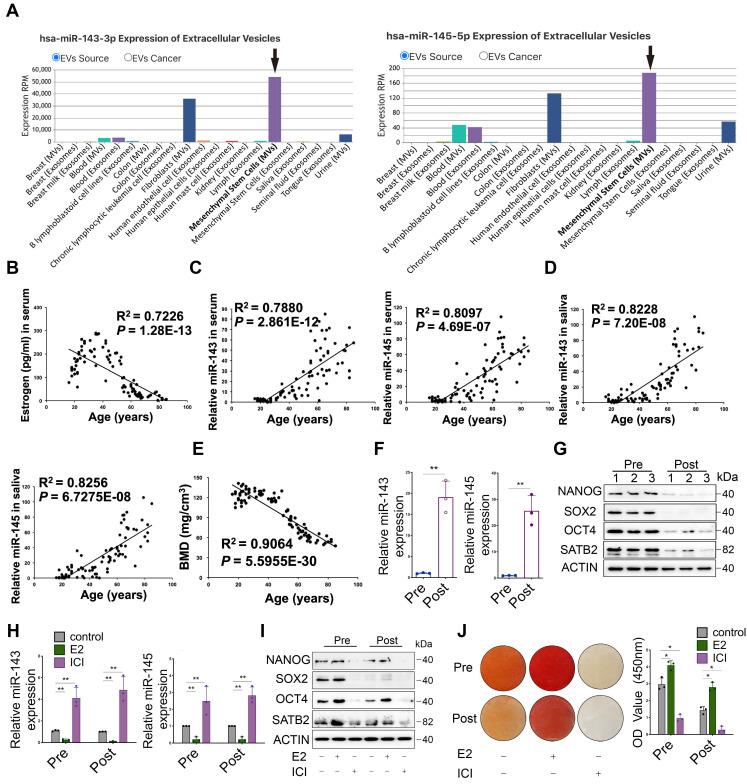
** Increases in miR-143/145 levels during estrogen-deficient osteoporosis and the attenuation of BMSCs function. (A)** The EVs miRNA database showed the expression of miR-143/145 in different sources. **(B)** Pearson correlation indicated the ELISA-detected levels of estrogen in 90 serum samples. **(C)** miRNA in the serum collected from 90 females were purified by miRNeasy Serum/Plasma Spike-In Control kit and analyzed for miR-143/145 detection by Pearson correlation. miR-143/145 expression were normalized to the spike-in control. **(D)** The Pearson correlation coefficient was analyzed for salivary miR-143/145 in the paired females. **(E)** The paired BMD value was calculated by CBCT to estimate the bone state. **(F)** Comparison of miR-143 and miR-145 in Pre- and Post-BMSCs. **(G)** The protein expression of NANOG, SOX2, OCT4 and SATB2 were detected in Pre- and Post-groups, which derived from 3 independent individuals, respectively. **(H)** The expression of miR-143 and miR-145 in estrogen or ICI treated BMSCs by qRT-PCR. **(I)** BMSCs stimulated with estrogen or ICI were analyzed for core TFs and SATB2 markers by Western blot. **(J)** Calcified nodules in estrogen or ICI stimulated BMSCs. Results are presented as the mean ± S.D. *p < 0.05; **p < 0.01; #*p* > 0.05 by Student's t test and one-way ANOVA.

To identify changes in miRNA expression during periods of decreasing estrogen levels, BMSCs from premenopausal and postmenopausal women were performed for miRNA microarray analysis, illustrating miR-143/145 were notably higher in postmenopausal BMSCs (Figure S1A). We further analyzed the database (http://bioinfo.life.hust.edu.cn/EVmiRNA) and found that across a diverse range of cells and tissues, miR‑143/145 loaded within EVs are mainly released by MSCs (Figure 1A). This suggests a potential correlation between miR‑143/145 levels and MSCs in the regulation of systemic diseases such as osteoporosis. Estrogen and miR-143/145 levels in 90 serum samples from premenopausal and postmenopausal women were further analyzed, and results showed that estrogen levels dramatically declined in 50~55 years of age (Figure 1B). In contrast, miR‑143/145 levels rose remarkably as estrogen levels dropped (Figure 1C). Salivary expression of miR-143/145 was also consistently higher in estrogen deficient samples (Figure 1D), indicating that salivary miR-143/145 testing may have potential diagnostic value for postmenopausal osteoporosis. Paired bone mineral density (BMD) values also indicated a significant decrease with estrogen dropping (Figure 1E). To more precisely investigate the skeletal microstructure, we obtained alveolar bone samples extracted during dental implant surgeries. Although there were no obvious differences between cortical bone comparisons, trabecular bone from postmenopausal women showed decreased volume, numbers, thickness, BMD, as well as increased trabecular separation relative to control (Figure S1B, C, D). Histomorphometric assays displayed the microstructural characteristics of osteoporosis, reduced osteoblast counts (Figure S1E) and increased osteoclast numbers (Figure S1F).

To further delineate the relationship between miR-143/145 and BMSCs function, we obtained BMSCs from premenopausal and postmenopausal donors and found that compared with premenopausal (Pre-) group, BMSCs from the postmenopausal (Post-) group displayed significantly increased miR‑143/145 (Figure 1F) and decreased clones (Figure S1G). Consistently, core TFs including NANOG, SOX2, OCT4 significantly reduced in Post-BMSCs (Figure S1H), as well as a DNA-binding protein, SATB2, emerging as a transcription factor regulator for osteoblast differentiation (Figure S1I). Similar results were also determined by qRT-PCR (Figure S1J) and Western blot assays (Figure 1G). We then examined the senescence state of BMSCs and found that γH2AX foci (Figure S1K) and SA-β-gal positive staining (Figure S1L) were enriched in Post-BMSCs relative to Pre-BMSCs. To further investigate the effect of estrogen on BMSCs function, we performed either estrogen or estrogen receptor antagonist (ICI 182, 780) stimuli on BMSCs and found that ICI significantly elevated the miR-143/145 expression (Figure 1H). Similarly, compared with the controls, colony formation (Figure S1M), core TFs (Figure 1I) and mineralized nodules (Figure 1J) were significantly reduced in ICI-treated BMSCs; correspondingly, estrogen improved these properties of BMSCs. Additionally, counts of SA-β-gal positive cells were remarkably higher in the ICI-treated group, while estrogen reversed this effect (Figure S1N). Taken together, these findings suggest that loss of estrogen in BMSCs led to increased miR-143/145 expression and impaired self-renewing and osteogenic differentiation.

### ERβ activates MIR143HG transcription to regulate the function of miR-143/145 that target core TFs and SATB2

**Figure 2:**
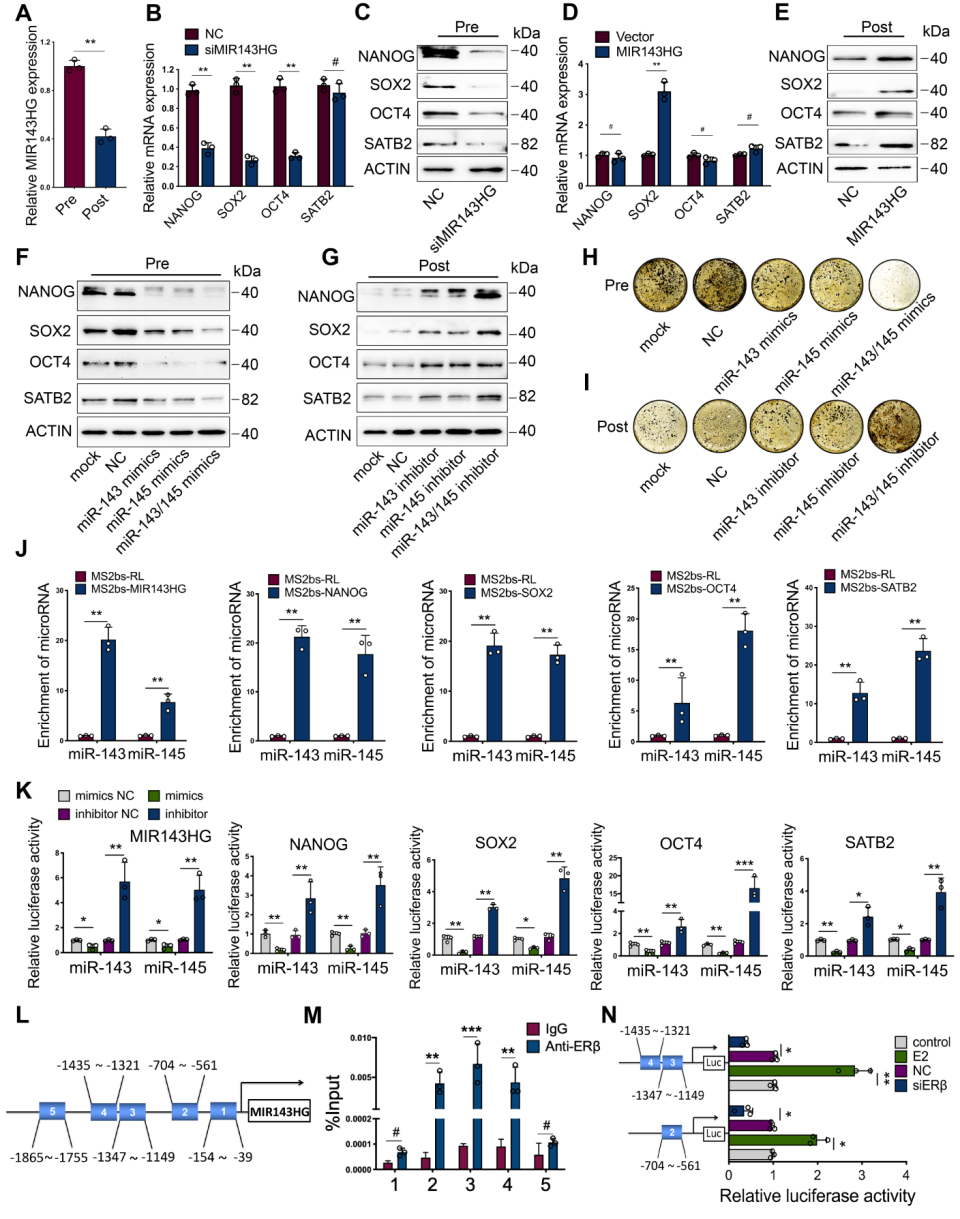
** ERβ activates MIR143HG transcription to regulate the function of miR-143/145 that target core TFs and SATB2. (A)** qRT-PCR showing the MIR143HG expression in Pre- and Post-BMSCs. **(B, C)** mRNA and protein expression of NANOG, SOX2, OCT4 and SATB2 were analyzed by qRT-PCR and Western blot in BMSCs transfected with siRNA targeting MIR143HG or negative control (NC). **(D, E)** mRNA and protein expression were analyzed in BMSCs transduced with MIR143HG overexpressing plasmid or control vector. **(F, G)** Protein levels of NANOG, SOX2, OCT4 and SATB2 in Pre- and Post-BMSCs were exacerbated by miR-143/145 overexpression and rescued by miR-143/145 inhibition by Western blot analysis. **(H, I)** Von Kossa staining showing the calcified nodules in Pre- and Post-BMSCs transfected with miR-143/145 mimics or inhibitor. **(J)** The transcripts including MIR143HG, NANOG, SOX2, OCT4 and SATB2 were specifically precipitated by MS2bp-GFP and the immunoprecipitated miRNAs were subjected to qRT-PCR analysis. MS2bs-RL was calculated as controls. RL, Renilla luciferase. **(K)** Luciferase reporters were included as MIR143HG, NANOG, SOX2, OCT4 and SATB2, and luciferase activity in 293T cells were co-transfected with miR-143/145 mimics or inhibitor. **(L, M)** ChIP analysis of ERβ binding to the MIR143HG promoter using five primers as predicted and the binding sites of region 2, 3, 4 were identified. Data are presented as the relative ration of ChIP to input. **(N)** According to ChIP analysis, three luciferase reporters were constructed and co-transduced into 293T cells with E2 and siERβ. E2, estrogen. Results are presented as the mean ± S.D. *p < 0.05; **p < 0.01; ***p < 0.001; #*p* > 0.05 by Student's t test and one-way ANOVA.

Since MIR143HG was reported to play a key role in regulating miR-143/145 function 19, we explored whether estrogen deficiency could influence its expression. We found that the basal level of MIR143HG was remarkably lower in Post-BMSCs than in Pre-BMSCs (Figure 2A). ICI significantly suppressed the MIR143HG expression, but estrogen obviously promoted its expression (Figure S2A). Considering that estrogen regulates downstream signaling pathways via binding with either ERα or ERβ, we first analyzed the effect of ERs on miR-143/145 and MIR143HG expression. Consequently, inhibition of ERβ, but not ERα, significantly influenced miR-143/145 (Figure S2B) and MIR143HG expression (Figure S2C). Next, we repressed MIR143HG expression with small interfering RNAs (siRNAs) and enhanced MIR143HG levels with plasmids overexpressing MIR143HG (Figure S2D, E). We found that MIR143HG inhibition in Pre-BMSCs markedly reduced core TFs, but there was no significant change in SATB2 (Figure 2B); however, protein levels for all of these factors declined (Figure 2C). Interestingly, after transfection with MIR143HG-overexpression vectors, only SOX2 mRNA levels increased significantly (Figure 2D), whereas protein levels for both core TFs and SATB2 were elevated (Figure 2E). To investigate the role of miR-143/145 in regulating core TFs and SATB2, Pre-BMSCs transfected with miR-143 and/or miR-145 were showed with a decreased protein levels (Figure 2F), while Post-BMSCs treated with miR-143 and/or miR-145 inhibitor displayed opposite results (Figure 2G). Of note, BMSCs co-transfected with miR-143/145 mimics or inhibitor showed a more significant alternation compared with a single miRNA transfection. Additionally, we explored the effect of miR-143/145 on osteogenesis and found that treating BMSCs with miR-143/145 mimics resulted in reduced of mineralized nodule formation, whereas BMSCs transfected with a miR-143/145 inhibitor showed continuous nodule enhancement (Figure 2H, I and Figure S2F, G).

To determine the direct binding interrelation between miR-143/145 and MIR143HG, as well as NANOG, SOX2, OCT4 and SATB2, RNA immunoprecipitation (RIP) was performed to pull down endogenous miR-143/145 by MS2 system, in which MS2 binding protein (MS2bp) specifically binding MS2 binding site (Figure S2H). Moreover, AGO2 was detected in RNA-protein complexes to determine the recruitment of AGO2 in RISC (Figure S2I). Complexes containing transcripts and miRNAs were immunoprecipitated with GFP antibody for subsequent qRT-PCR. Using MS2bs-Renilla luciferase (RL) as control, we found high miR‑143/145 enrichment in MS2-MIR143HG binding RNAs. Similar miR‑143/145 enrichment results were observed in NANOG, SOX2, OCT4, SATB2 transcripts (Figure 2J). To further elucidate direct binding relationships, we constructed dual-luciferase reporters and found that miR‑143/145 mimics reduced the luciferase activity of reporter vectors containing MIR143HG, NANOG, SOX2, OCT4 or SATB2 relative to NC treatment, whereas miR‑143/145 inhibitors enhanced luciferase activity for all five vectors (Figure 2K and Figure S2J). Taking into consideration both the role of miR-143/145 and MIR143HG in regulating BMSCs pluripotent genes and our findings that ERβ induces significant changes in MIR143HG expression, we investigated the mechanism by which ERβ regulates MIR143HG. We first analyzed for putative ER binding sites in the MIR143HG promoter and found that 5 binding regions are predicted for ERβ, but not ERα. ChIP assays were performed based on CUT&Tag techniques and 5 primers were constructed to cover these five regions (1:-154~39, 2:-704~-561, 3:-1347~-1149, 4:-1435~-1321, 5:-1865~-1755) by specific ERβ antibody (Figure 2L). The qRT-PCR results showed significant enrichment at regions 2, 3, and 4, but no difference at regions 1 and 5 (Figure 2M), which was also evidenced by ChIP gel shift assay (Figure S2K). For further binding site verification, we constructed luciferase reporters containing regions 2, 3, and 4 and found that luciferase activity of these reporters increased with estrogen stimulation and decreased with siERβ transfection (Figure 2N). Overall, these findings suggest that ERβ activates MIR143HG transcription, which in turn couples with miR-143/145 via a ceRNA mechanism to mediate core TFs and SATB2 expression.

### miR-143 and MIR143HG complexes shuttled into nuclei and cooperatively regulates SOX2 transcription

**Figure 3:**
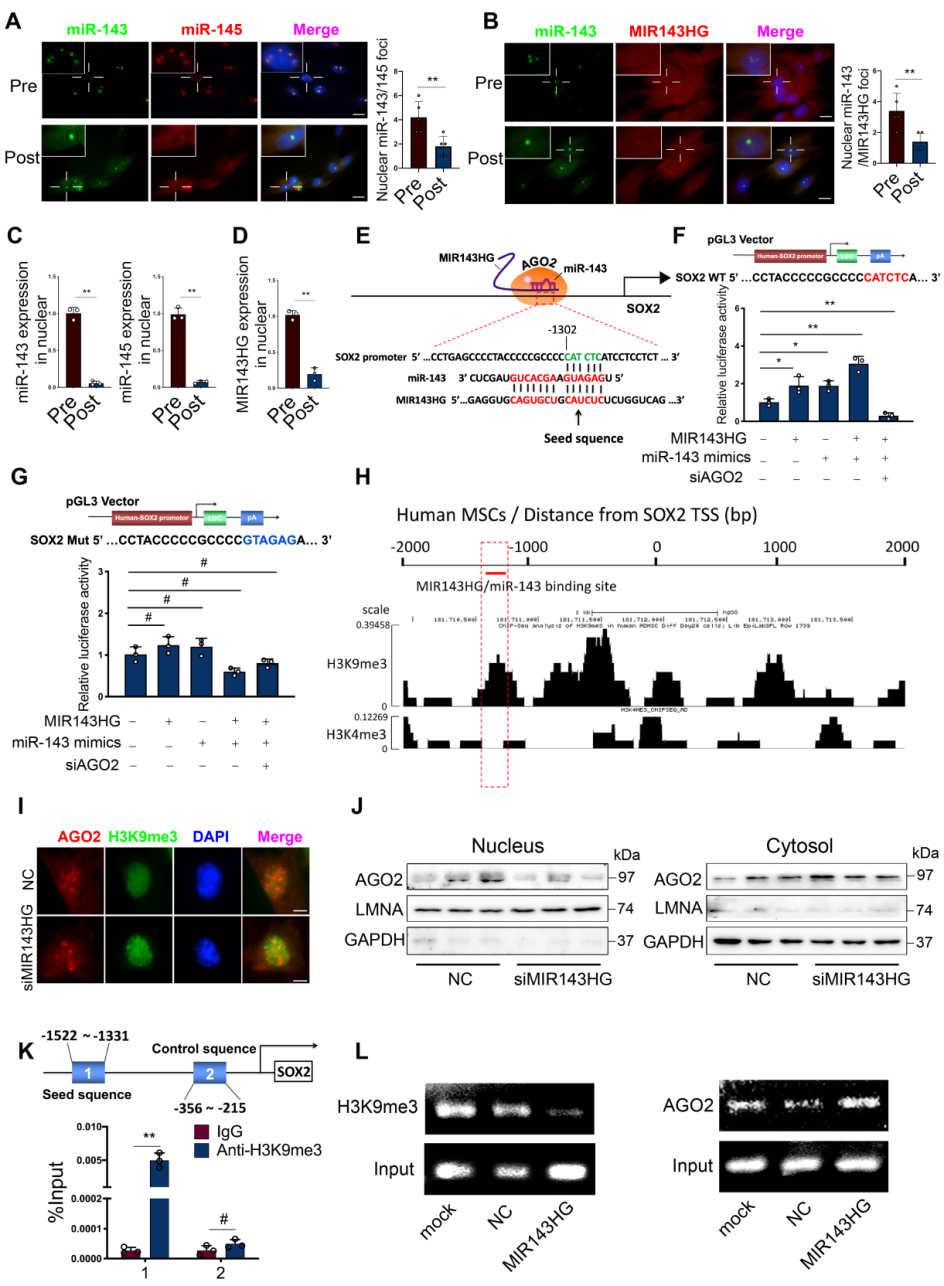
** miR-143 and MIR143HG complexes shuttled into nuclei and cooperatively regulates SOX2 transcription. (A)** Nuclear and cytoplasmic distribution of miR-143 and miR-145 in Pre- and Post-BMSCs as labelled by FISH assay. Scale bars: 20 μm. **(B)** Nuclear and cytoplasmic localization of MIR143HG and miR-143 in Pre- and Post-BMSCs. Scale bars: 20 μm. **(C)** qRT-PCR analysis for miR-143 and miR-145 in BMSCs nuclei. U6 was used as endogenous control. **(D)** qRT-PCR analysis for MIR143HG in BMSCs nuclei. GAPDH was used as endogenous control. **(E)** A predicted region of SOX2 promoter by sequence alignment was selected as recognition sites by MIR143HG and miR-143 complex. **(F)** Wild type plasmid based on predicted region of SOX2 promoter was subjected to dual-luciferase reporter assay in response to MIR143HG-overexpressing vector, miR-143 mimics and siAGO2. **(G)** Mutant plasmid based on the predicted region was subjected to dual-luciferase reporter assay in response to MIR143HG-overexpressing vector, miR-143 mimics and siAGO2. **(H)** Enrichment of H3K9me3, and H3K4me3 on the SOX2 promoter from Cistrome database. **(I)** After addition of siMIR143HG, AGO2 and H3K9me3 were examined by IF. Scale bars: 4 μm. **(J)** AGO2 isolated from nuclei and cytoplasm were detected by western blot in response to siMIR143HG treatment. **(K)** ChIP analysis of H3K9me3 binding to the targeted region by MIR143HG and miR-143 complex. **(L)** After transfected with mock, NC and MIR143HG overexpressing vector, BMSCs were subjected to ChIP assay by H3K9me3 or AGO2 antibody. The precipitated and amplified DNA were performed to gel shift assay. Input DNA were conducted as a control. Results are presented as the mean ± S.D. *p < 0.05; **p < 0.01; #*p* > 0.05 by Student's t test and one-way ANOVA.

To confirm our qRT-PCR results (Figure 1F) and further delineate the subcellular localization of miR-143/145, we used FISH assays to examine their expression in Pre- and Post‑BMSCs. Surprisingly, we found that miR-143 and miR-145 from Pre-BMSCs existed in similar spatial positions and mostly assembled in nuclei but not in the cytoplasm; however, in Post-BMSCs, the number of fluorescent miR‑143 and miR‑145 foci decreased in nuclei and the majority were observed in cytoplasm (Figure 3A). Also, we examined MIR143HG and miR-143 localization in Pre- and Post-BMSCs, which showing that MIR143HG and miR-143 were co-localized in nuclei in Pre-BMSCs, but the foci numbers were decreased in Post-BMSCs (Figure 3B). Next, to further confirm the distribution characteristics of miR-143/145 and MIR143HG in BMSCs, nuclear and cytoplasmic RNA were isolated for qRT-PCR assay (Figure 3C, D), also indicating a decreased expression of miR-143/145 and MIR143HG in nuclei from Post-BMSCs. Since miR-143/145 and MIR143HG were restricted to one location, we hypothesized a possibility that MIR143HG might induce miR-143/145 into nuclei and co-regulate gene transcription. This hypothesis might be partially underpinned by a previous study in which small nuclear double-stranded RNA (dsRNA) activated gene expression by targeting gene promoters in a process labeled dsRNA-induced gene activation (RNAa) 20. Successful RNAa is dependent on several rules, as follows: (i) a preferred dsRNA length of 21nt dsRNA (miR‑143: 21nt; miR‑145: 23nt); (ii) a 5` dsRNA antisense strand or seed sequence matching the promoter is important to initiate transcriptional activation, as the 5` end of miR‑143 is highly matched to MIR143HG; (iii) RNAa requires Ago2 protein indispensably that involves in demethylated histone 3 at lysine-9 (H3K9me3) to activate transcription. Therefore, we investigated whether the MIR143HG‑miR‑143/145 complex matches the promoter region of core TFs and SATB2, for which transcription could be influenced by MIR143HG and miR‑143/145. Consequently, we only found one putative region on the SOX2 promoter that matched the MIR143HG and miR-143 complex according to the RNAa rules (Figure 3E). Moreover, SOX2 mRNA levels, but not other pluripotent genes, increased after overexpressing MIR143HG (Figure 2D), further indicate that the complexes may regulate SOX2 transcription. To verify our hypothesis, we cloned the predicted SOX2 promoter region into pGL3 plasmids for subsequent dual-luciferase reporter assays. When transfected with MIR143HG-overexpressing vector or/and miR‑143 mimics, luciferase activity increased compared with the counterpart. However, when siAGO2 was transfected upon addition of the MIR143HG-overexpressing vector and miR‑143 mimics, luciferase activity reduced significantly (Figure 3F). We also constructed a mutation to the predicted region and found that the altered luciferase activity of MIR143HG-overexpressing vector, miR-143 mimics and siAGO2 were abrogated by co-transfected with mutated plasmid compared with WT mutation (Figure 3G). Cistrome database were analyzed to detect the enrichment of histone methylation on the SOX2 promoter in human MSCs. Interestingly, we observed a high enrichment of H3K9me3 at MIR143HG and miR-143/145 complex binding site, but low H3K4me3 enrichment throughout the SOX2 promoter (Figure 3H). To corroborate the effect of complexes on H3K9me3 to regulate chromatin state, IF analysis suggest that upon transduction with siMIR143HG, AGO2 levels fused into nucleus were significantly reduced, but H3K9me3 levels were remarkably enhanced (Figure 3I). We further assessed the AGO2 distribution with Western blot and obtain the similar results (Figure 3J). To determine whether the MIR143HG and miR-143 complex controls SOX2 transcription by modulating H3K9me3 recruitment, we designed primers (1: -1522~-1331, seed sequence; 2: -356~-215, control sequence) and performed ChIP assay using anti-H3K9me3 antibody. We found that SOX2 primer 1, but not primer 2, resulted in a significant enrichment (Figure 3K) and the amplified products were collected and verified by ChIP gel shift assay (Figure S3A). Then, cells were transfected with MIR143HG overexpression vectors for ChIP assays using primer 1 to determine the changes of H3K9me3 and AGO2. Compared with mock and NC transfection, a pronounced decline in H3K9me3 and significant increase in AGO2 (Figure 3L and Figure S3B, C) occurred after MIR143HG overexpression transfection. Taken together, our above findings suggest that nuclear transfer of miR-143 and MIR143HG complexes activate SOX2 transcription by opening promoter chromatin via an RNAa mechanism.

### Depletion of miR-143/145 in mice prevents bone loss and retains bone regeneration in OVX-induced osteoporosis

**Figure 4:**
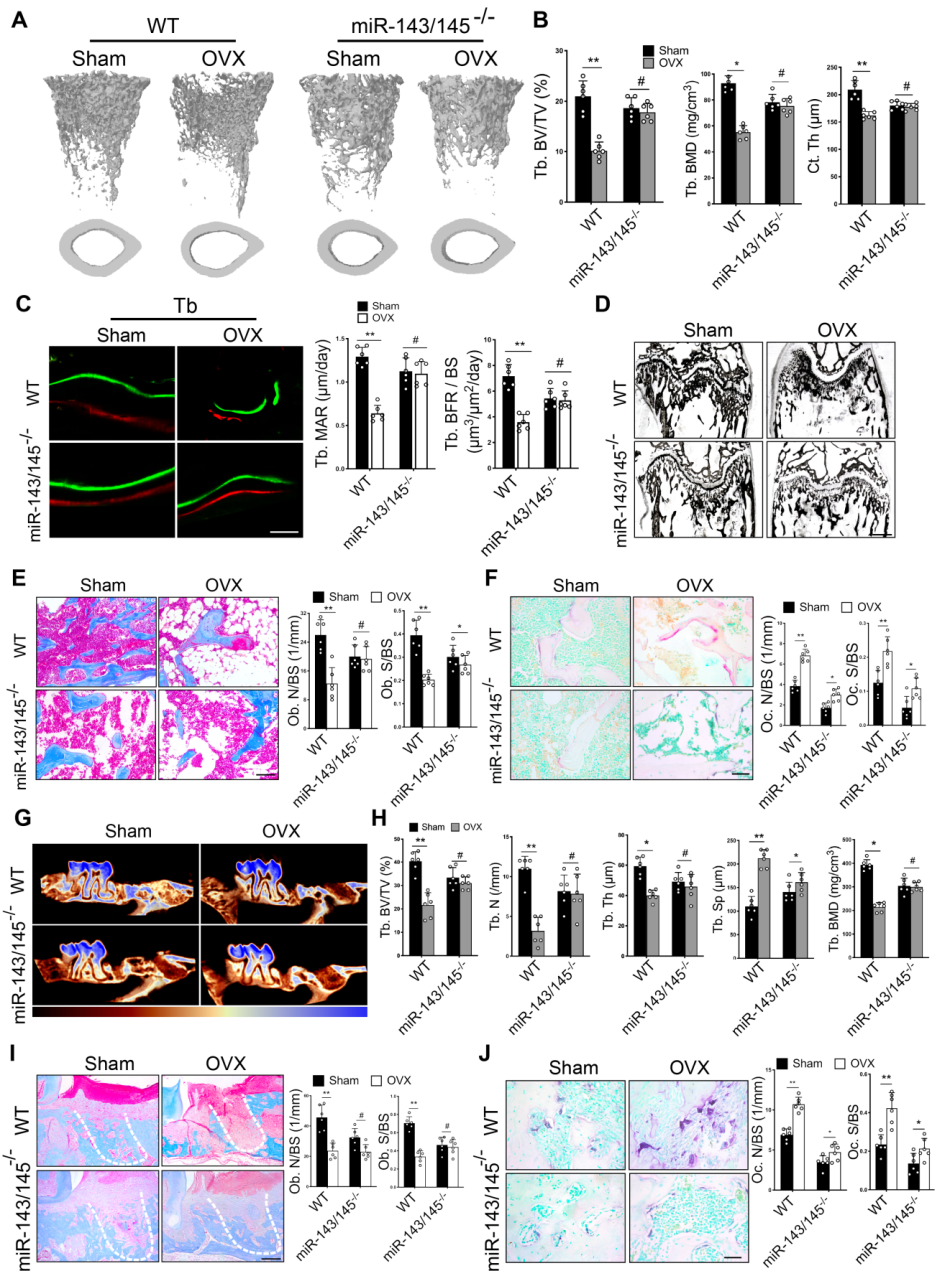
** Depletion of miR-143/145 in mice prevents bone loss and maintains bone regeneration in OVX-induced osteoporosis. (A)** Micro-CT analysis of trabecular and cortical bone in WT and *miR-143/145^-/-^* mice following OVX. **(B)** Quantitative measurements of Tb. BV/TV, Tb. BMD and Ct. Th. **(C)** Representative images of dynamic histomorphometry of trabecular bone (Tb) with quantification of mineralization apposition rate (MAR) and bone formation rate per bone surface (BFR/BS). Scale bars: 20 μm. **(D)** Representative Von Kossa staining showing an overall trabecular bone mass of WT and* miR-143/145^-/-^* mice following Sham and OVX operation. Scale bars: 500 μm. **(E)** Representative masson trichrome staining showing trabecular bone accounts and quantification of osteoblast numbers and surface on the trabecular. Scale bars: 100 μm. **(F)** Representative TRAP staining showing osteoclastic activity and quantification of osteoclast numbers and surface on the trabecular. Scale bars: 50 μm. **(G)** Micro-CT analysis of new bone fill in tooth extraction socket by color coded thickness maps. Color changes from red to blue denote a gradual elevation in bone thickness. **(H)** Quantitative measurements of Tb. BV/TV, Tb. N, Tb. Sp, Tb. Th, and Tb. BMD for new bone in extraction socket. **(I)** Representative masson trichrome staining showing the new bone mass and osteoblast numbers and surface on the trabecular in tooth extraction socket, which was delineated by white dotted lines. Scale bars: 100 μm. **(J)** Representative TRAP staining showing osteoclastic activity and quantification of osteoclast numbers and surface on the trabecular in extraction socket. Scale bars: 50 μm. Results are presented as the mean ± S.D. *p < 0.05; **p < 0.01; #*p* > 0.05 by two-way ANOVA, n = 6 mice per group.

We next investigated the roles of miR‑143/145 *in vivo*. A miR‑143/145 knockout mouse model was used to explore whether miR-143/145 deficiency could prevent osteoporotic bone loss. We genotyped *miR-143/145^-/-^* mice by PCR (Figure S4A) and found that there was no significant difference in skeletal alternation between WT and *miR-143/145^-/-^* newborn mice (Figure S4B), consistent with a previous study 18. Then, we established ovariectomized (OVX) models in 3-month-old WT and *miR-143/145^-/-^* mice and observed distal femurs after 2 months. Micro-CT analysis suggested that OVX WT mice had a significant reduction in bone mass compared with Sham-operated (control) mice; however, *miR-143/145^-/-^* mice following OVX did not show any notable decrease compared with the control group (Figure 4A). Trabecular thickness maps showed a significant decline of bone thickness in OVX-induced WT mice, but only a slight decline in OVX-induced *miR-143/145^-/-^* mice compared with the control *miR-143/145^-/-^* mice (Figure S4C). Meanwhile, trabecular bone volume, numbers, thickness, BMD and cortical thickness were markedly reduced in OVX-induced WT mice, whereas only a slight decrease of these bone parameters was observed in OVX-induced *miR-143/145^-/-^* mice compared to controls (Figure 4B and Figure S4D). Dynamic histomorphometry showed that OVX-induced WT mice had remarkably lower trabecular (Figure 4C) and endosteal bone formation rates (Figure S4E) compared with the control group, whereas this trend was almost completely absent in *miR-143/145^-/-^* mice. Von Kossa staining revealed that WT mice showed lower trabecular bone mass after OVX, but *miR-143/145^-/-^* mice showed no significant differences after OVX (Figure 4D). Masson trichrome staining and analysis displayed that estrogen deficiency led to a marked declined number of osteoblasts on the trabecular in WT mice, but not in *miR-143/145^-/-^* mice (Figure 4E). Measurement of OCN positive osteoblasts supported these findings (Figure S4F). To further assess the influence of miR-143/145 depletion on bone resorption during bone homeostasis, we examined osteoclastic activity by TRAP staining. Consequently, although the elevation was more pronounced in WT mice, the osteoclastic activity was also increased in *miR-143/145^-/-^* mice after OVX compared to the control* miR-143/145^-/-^* mice (Figure 4F). Thus, depletion of miR-143/145 could effectively prevent bone loss in OVX-induced osteoporosis.

To confirm the effect of miR-143/145 depletion on bone regeneration in OVX-induced mice, we established models of tooth extraction by removing the first molar in the respective mice groups. Two weeks after surgery, we found that new bone formation in extraction socket was significantly decreased in OVX-induced WT mice, whereas no significant difference was observed in OVX-induced* miR-143/145^-/-^* mice compared to the control (Figure 4G and Figure S4G). Trabecular bone volume, numbers, thickness, and BMD were lower, and the trabecular separation was higher in OVX-induced WT mice compared with controls; however, OVX only induced a slight change of bone parameters in *miR-143/145^-/-^* mice (Figure 4H). Consistently, histomorphometry showed that the amount of new bone fill in distal root socket was remarkably decreased in OVX-induced WT mice, but only a slight decrease was observed in comparison of Sham and OVX-induced *miR-143/145^-/-^* mice (Figure S4H). Additionally, OVX decreased osteoblast numbers and surface on the trabecular in WT mice, but not in *miR-143/145^-/-^* mice (Figure 4I). We next analyzed osteoclast activity and found that while there was a more significant increase in activity in OVX-induced WT mice, osteoclastic activity also rose in *miR-143/145^-/-^* mice compared with the Sham counterparts (Figure 4J). From this, we concluded that depletion of miR‑143/145 was helpful to maintain bone regeneration ability in OVX-induced mice.

### miR-143/145 depletion counteracts the adverse effects of estrogen deficiency on BMSCs function

**Figure 5:**
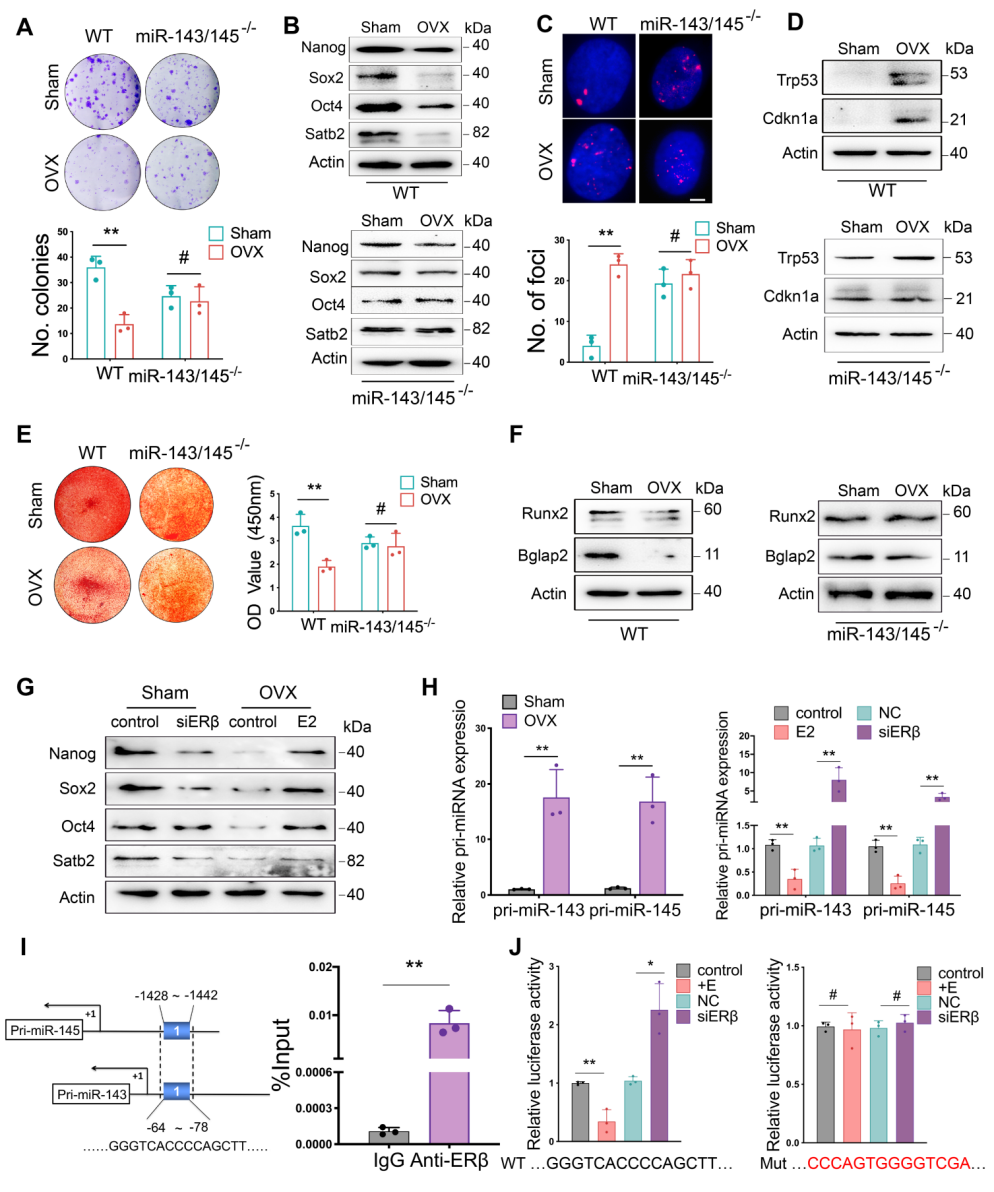
** miR-143/145 depletion counteracts the adverse effects of estrogen deficiency on BMSCs function. (A)** CFU analysis of colonies in Sham and OVX-induced WT and *miR-143/145^-/-^* BMSCs. Lower panel shows the respective quantification. **(B)** Western blot revealing the protein expression of Nanog, Sox2, Oct4, and Satb2.** (C)** γH2AX staining showing the senescence difference in WT and *miR-143/145^-/-^* BMSCs following OVX. Lower panel shows the respective quantification. Scale bars: 4 μm. **(D)** Western blot revealing the protein expression of Trp53 and Cdkn1a. **(E)** Alizarin red revealing the alternation of osteogenic differentiation. Right panel shows the respective quantification. **(F)** Western blot revealing osteogenic markers of Runx2 and Bglap2 and** (G)** core TFs and SATB2. **(H)** The expression of pri-miR-143/145 was increased in OVX-induced BMSCs and in estrogen or siERβ treated BMSCs. **(I)** ChIP analysis of ERβ binding to the shared putative region of pri-miR-143 and pri-miR-145 promoter. **(J)** WT and Mutation vectors were constructed for dual-luciferase reporter assay and co-transfected with estrogen or siERβ. Results are presented as the mean ± S.D. *p < 0.05; **p < 0.01; #*p* > 0.05 by Student's t test and one-way ANOVA.

To explore the effect of miR-143/145 depletion on the self-renewing and osteogenic differentiation of mouse BMSCs, we isolated BMSCs from WT and *miR-143/145^-/-^* mice following OVX. Colonies and proliferation of BMSCs significantly declined in WT group following OVX, but no apparent differences were observed between the Sham and OVX *miR‑143/145^-/-^* groups (Figure 5A and Figure S5A). Consistent with these results, an obvious reduction of Nanog, Sox2, Oct4 and Satb2 levels were observed in WT BMSCs following OVX, but not in *miR-143/145^-/-^* BMSCs (Figure 5B). Moreover, the senescence was significantly greater in OVX-induced WT BMSCs as indicated by SA-β-gal and γH2AX staining, as well as Trp53 and Cdkn1a expression. However, no such effect was observed for *miR-143/145^-/-^* BMSCs between Sham and OVX group (Figure 5C, D and Figure S5B). We further delineated the protective impact of miR‑143/145 on lineage differentiation of OVX BMSCs, while an obvious reduction of osteogenesis (Figure 5E, F) and elevation of adipogenesis in WT BMSCs after OVX (Figure S5C). These data indicate that miR‑143/145 depletion can counteract the adverse effects of estrogen deficiency on BMSCs function.

We next investigated the effect of estrogen on miR-143/145 expression and core TFs in Sham and OVX-induced WT BMSCs. As previous results of human BMSCs, decreased miR-143/145 expression and increased TFs in mouse BMSCs were observed by estrogen treatment, whereas this effect was reversed by siERβ treatment (Figure 5G and Figure S5D). To further analyze whether ERβ transcriptionally influences miR-143/145 expression, we examined primary (pri-) transcripts of miR-143/145. In line with miR-143/145 expression, similar changes of pri-miR-143/145 were observed in OVX-induced BMSCs and in estrogen or siERβ treated BMSCs (Figure 5H), which suggesting that ERβ might regulate miR-143/145 expression at transcription level. Thus, we analyzed the putative binding sites by ERs in pri-miR-143/145 promoter and found one putative binding region located in both the pri-miR‑143 and pri-miR‑145 promoters was predicted for ERβ, but not ERα. Then, we designed the primers accordingly and performed ChIP assay using ERβ antibody. Consequently, a significant enrichment was observed by qRT-PCR compared with the control IgG (Figure 5I). Further, we constructed luciferase reporters based on the predicted region and found that luciferase activity with WT vectors was decreased by estrogen treatment and increased by siERβ transfection, whereas this effect was eliminated in assays using mutant vectors (Figure 5J). These findings suggest that ERβ could simultaneously inhibit transcription of miR-143 and miR-145, which in turn affects BMSCs function.

### EVs loaded miR-143/145 from BMSCs activates osteoclast function

**Figure 6:**
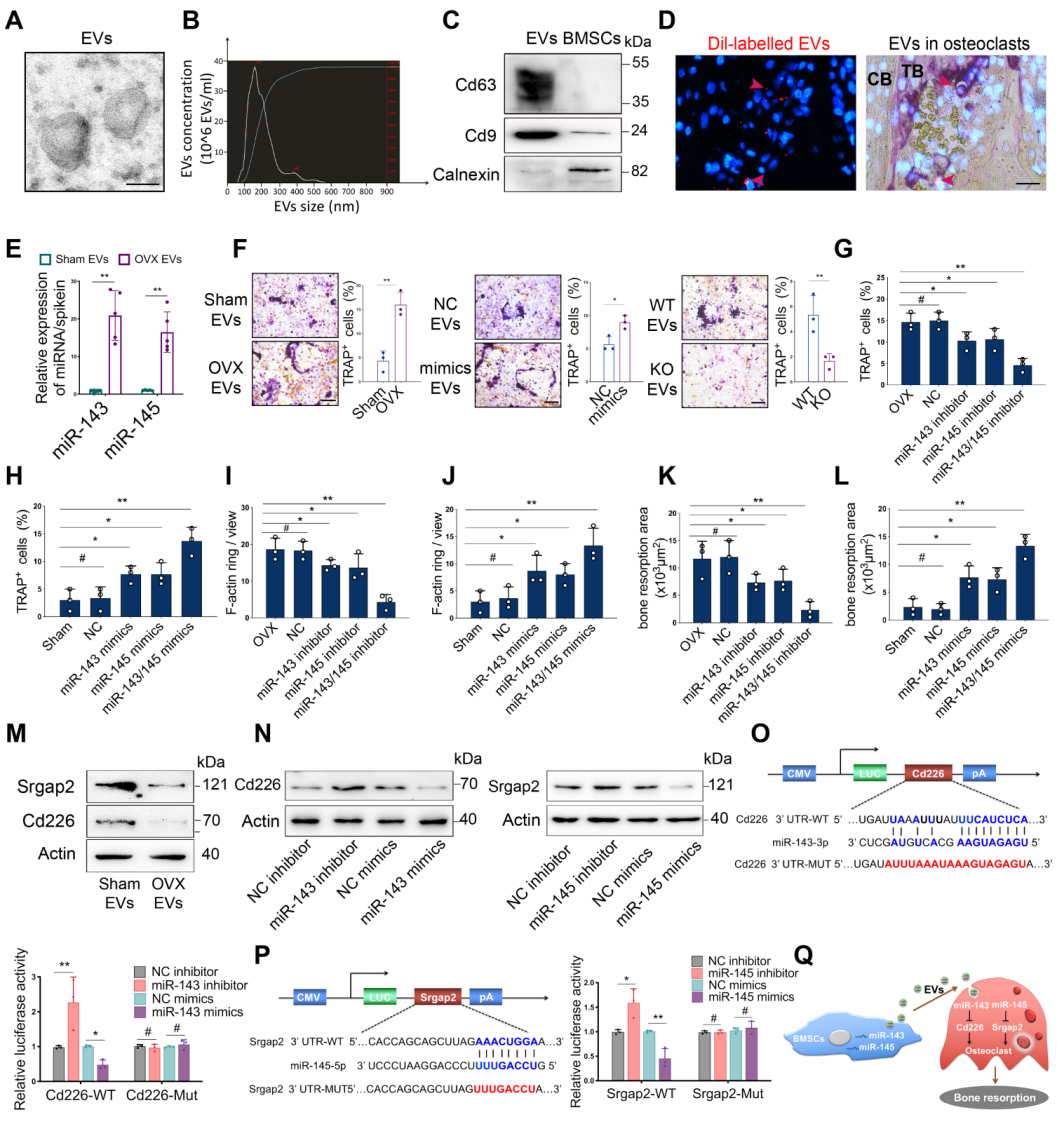
** EVs loaded miR-143/145 from BMSCs activates osteoclast function. (A)** A representative image of EVs from BMSCs by transmission electron microscopy (TEM). Scale bars: 100 nm. **(B)** NTA of EVs for calculating the size distribution. The left Y-axis indicated particle number concentration and X-axis indicated particle size. **(C)** Cd63, Cd9, and Calnexin from EVs and BMSCs were detected by Western blot. **(D)** Fluorescent microscopy and TRAP staining analysis revealing DiI-labeled EVs injected into bone marrow. Red arrow head indicated DiI-labeled EVs. Scale bars: 50 μm. **(E)** miR-143/145 loaded in EVs from Sham and OVX-induced BMSCs were examined by qRT-PCR.** (F)** EVs from OVX BMSCs, miR-143/145 mimics treated BMSCs or *miR-143/145^-/-^* BMSCs were co-cultured with osteoclasts and visualized by TRAP staining. Scale bars: 100 μm. Quantitative measurements of** (G, H)** TRAP positive staining cells, **(I, J)** F-actin ring staining cells, and **(K, L)** bone resorption area in miR-143/145 inhibitor or mimics treated osteoclasts. **(M)** Western blot showing the Cd226 and Srgap2 protein levels in EVs treated osteoclasts. **(N)** Western blot showing the Cd226 and Srgap2 expression in miR-143/145 inhibitor or mimics treated osteoclasts.** (O, P)** WT and mutation of Cd226 or Srgap2 vectors were subjected to dual-luciferase reporter assays. **(Q)** A schematic working model for miR-143/145 shuttling from BMSCs to osteoclasts through EVs. Results are presented as the mean ± S.D. *p < 0.05; **p < 0.01; #*p* > 0.05 by Student's t test and one-way ANOVA.

Based on the observations that OVX-induced bone loss correlated with increased bone resorption and that the amount of BMSCs-derived EVs containing miR-143/145 were secreted into extracellular fluid or circulation in response to estrogen deficiency, we investigated whether miR-143/145 are involved with osteoclast function. The microstructure of EVs derived from the supernatants of mouse BMSCs cultures was verified by TEM (Figure 6A and Figure S6A). The size distribution of EVs was calculated by nanoparticle tracking analysis (NTA) (Figure 6B and Figure S6B). Western blots showed that EVs highly expressed the characteristic proteins Cd63 and Cd9 and minimally expressed Calnexin, a cytosolic marker protein (Figure 6C). EVs incorporation into osteoclasts was confirmed using DiI-labelling of EVs by co-cultured with osteoclasts (Figure S6C) and injected into bone marrow (Figure 6D). Analysis by qRT-PCR showed that EVs derived from OVX BMSCs had greater miR-143/145 levels than Sham BMSCs (Figure 6E) and osteoclasts treated with OVX EVs also showed increased miR-143/145 levels (Figure S6D). EVs from OVX BMSCs and miR-143/145 mimics treated BMSCs drastically increased multinucleated osteoclasts, while *miR-143/145^-/-^* BMSCs derived EVs impaired osteoclast differentiation compared to the counterparts (Figure 6F).

Then we further investigated the role of miR-143/145 in osteoclast function, and found miR-143 and/or miR-145 inhibitor remarkably reduced multinucleated osteoclasts, impaired F-actin ring formation and suppressed bone resorption (Figure 6G, I, K and Figure S6E); however, miR-143 and/or miR-145 mimics produced the opposite effect (Figure 6H, J, L and Figure S6F). We used bioinformatic analysis to search for miR-143 and miR-145 targeting candidates that were associated with induction of osteoclastogenesis. Results predicted that Cd226, also called DNAM-1 (DNAX accessory molecule-1), which inhibits osteoclast differentiation by regulating cellular fusion process 21, is the direct target of miR-143 and that SLIT ROBO Rho GTPase-activating protein 2 (Srgap2), which represses osteoclast formation by modulating cytoskeletal organization 22, is the direct target of miR-145. We subsequently analyzed Cd226 and Srgap2expression and found both were reduced in osteoclasts treated with supernatant from OVX BMSCs (Figure 6M). Moreover, transfecting either miR-143 inhibitors or mimics into osteoclasts resulted in significantly increased and decreased, respectively, Cd226 expression. Similar trends were also observed for Srgap2 expression with miR-145 inhibitor or mimics treatment (Figure 6N). To further confirm whether miR-143 or miR-145 directly binds to the 3`UTRs of Cd226 or Srgap2, we constructed WT and mutation vectors for dual-luciferase reporter assays. Indeed, miR-143 inhibitors or mimics drastically influenced luciferase activities when co-transfected with WT vectors, but mutation vectors thoroughly abolished this effect (Figure 6O). Similar results were also found in Srgap2 WT or mutation vectors (Figure 6P). Collectively, these data suggest that EVs loaded miR-143/145 from BMSCs could transfer to osteoclasts to affect osteoclast activity and function (Figure 6Q).

### AntagomiR-143/145 prevents and agomiR-143/145 exacerbates OVX-induced bone loss

**Figure 7:**
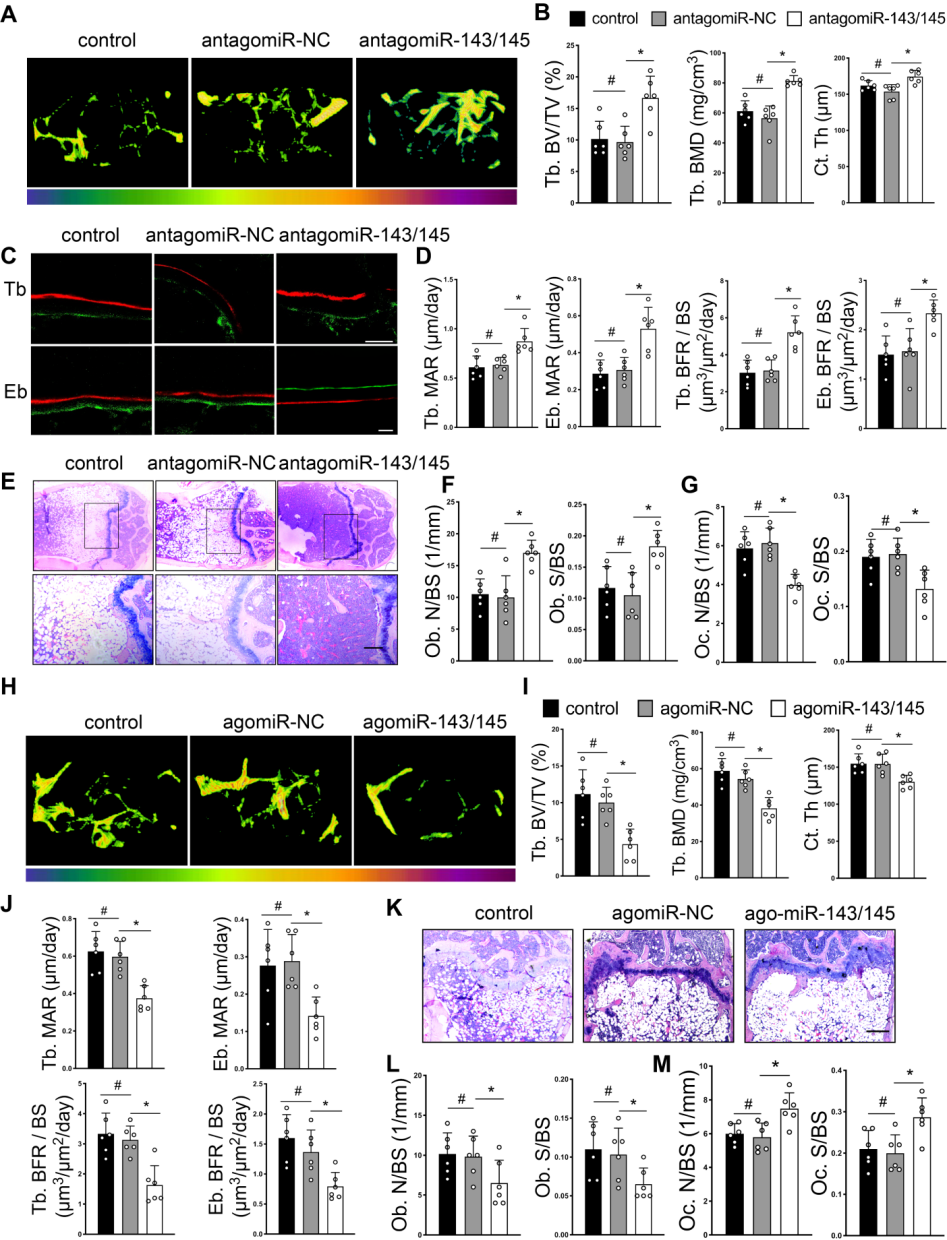
** AntagomiR-143/145 prevents and agomiR-143/145 exacerbates OVX-induced bone loss. (A)** Representative micro-CT images of trabecular and cortical bone from control, antagomiR-NC, and antagomiR-143/145 treated femurs. **(B)** Quantitative measurements of Tb. BV/TV, Tb. BMD and Ct. Th. **(C)** Dynamic histomorphometry analysis of Tb and Eb bone including the quantification **(D)** of MAR and BFR/BS. Scale bars: 20 μm. **(E)** HE staining showing an overall trabecular bone mass. Scale bars: 200 μm. **(F)** Quantification of osteoblast numbers and surface on the trabecular. **(G)** Quantification of osteoclast numbers and surface on the trabecular. **(H)** Micro-CT images of trabecular from control, agomiR-NC, and agomiR-143/145 treated mice. **(I)** Measurements of Tb. BV/TV, Tb. BMD and Ct. Th in respective femurs. **(J)** Quantification of MAR and BFR/BS in Tb and Eb bone. **(K)** Representative images of HE staining showing an overall trabecular bone mass. Scale bars: 200 μm. **(L)** Quantification of osteoblast numbers and surface on the trabecular. **(M)** Quantification of osteoclast numbers and surface on the trabecular. Results are presented as the mean ± S.D. *p < 0.05; #*p* > 0.05 by one-way ANOVA, n = 6 mice per group.

To explore therapeutic value of miR-143/145, we constructed and delivered antagomiR-143/145 into femoral bone marrow cavities of OVX-induced mice. Before injected with antagomiR-143/145 into the bone marrow, the efficiency of antagomiR targeting BMSCs and osteoclasts were confirmed by qRT-PCR (Figure S7A). Micro-CT analysis revealed greater trabecular bone mass and cortical bone thickness in antagomiR-143/145 treated mice compared with control or antagomiR-NC counterparts (Figure 7A and S7B). Trabecular bone volume, BMD and cortical thickness were increased and trabecular separation was decreased in femurs of antagomiR-143/145 injected mice (Figure 7B). Dynamic histomorphometry also showed that antagomiR-143/145 remarkably elevated trabecular and endosteal bone formation rates (BFR) compared with control or antagomiR-NC counterparts (Figure 7C, D). Additionally, HE and Von Kossa staining revealed that a considerable amount of trabecular bone was retained in antagomiR-143/145 treated mice (Figure 7E and S7C). Consistently, injection of antagomiR-143/145 contributed to a greater increase in osteoblasts numbers and surface on the trabeculae (Figure 7F and S7D), which was also validated by OCN positive osteoblasts (Figure S7E). Further, to examine whether miR-143/145 inhibition in bone marrow influence bone resorption, we performed TRAP staining and found that the amounts of osteoclasts were reduced in antagomiR-143/145-treated mice compared with the controls (Figure 7G and S7F).

Conversely, we investigated whether miR-143/145 overexpression aggravated OVX-induced bone loss by constructing and injecting agomiR‑143/145 into the bone marrow cavity. qRT-PCR analysis confirmed the efficiency of agomiR targeting BMSCs and osteoclast (Figure S7G). Compared with the controls, mice treated with agomiR-143/145 exhibited aggravated bone loss and declined cortical thickness (Figure 7H and S7H), which also confirmed by relative bone parameters (Figure 7I), and a decreased BFR of trabecular and endosteal bone (Figure 7J and S7I). Moreover, barely any trabecular bone was observed in agomiR-143/145 treated group by HE and Von Kossa staining (Figure 7K and S7J). Similarly, agomiR-143/145 led to a reduced osteoblasts numbers and surface by masson trichrome staining (Figure 7L and S7K), as well as OCN positive staining (Figure S7L). Furthermore, increased osteoclast numbers were observed in the femurs of agomiR-143/145-treated mice (Figure 7M and S7M). Taken together, these findings indicate that antagomiR-143/145 prevented OVX-induced bone loss while agomiR-143/145 exhibited an opposite effect.

## Discussion

**Figure 8:**
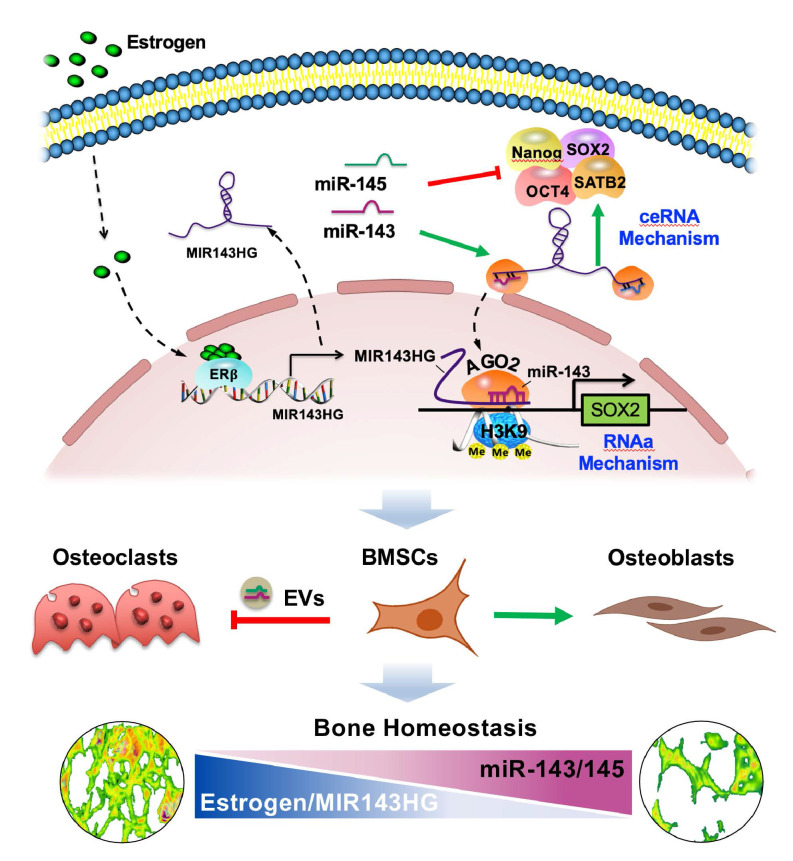
** Working model of miR-143/145 in regulating BMSCs stemness properties and osteoclast activity during progressive postmenopausal bone loss.** By investigating links between progressive postmenopausal bone loss and impaired BMSCs function, miR-143/145 cooperates with MIR143HG to regulate BMSCs pluripotency genes by protecting gene translation and activating SOX2 transcription. Inhibition of miR-143/145 could improve bone formation and reduce bone resorption in estrogen-deficient osteoporosis.

Our study extended miRNA function by determining that miR-143 cooperated with LncRNA MIR143HG to nuclear localization and influenced local chromatin structure of SOX2 promoter (Figure 8). At the molecular level, MIR143HG, regulated by ERβ, decoyed miR-143/145 to maintain the normal posttranslational activity of core TFs through canonical ceRNA mechanism. Then, the miR-143 and MIR143HG complexes bond to SOX2 promoter and enhanced its transcription by decreasing H3K9me3 accumulation. This noncanonical mechanism expands the known understanding of miRNA exerted posttranslational activity. At the cellular level, EVs loaded miR-143/145 from BMSCs were shuttled into osteoclasts and accelerated osteoclastic resorption. For therapeutic application, Loss of miR-143/145 attenuated OVX-induced osteoporosis, which suggests that miR-143/145 might be a target for postmenopausal osteoporosis. For diagnostic application, miR-143/145 are abundant in both serum and saliva samples from postmenopausal women, and non-invasive salivary detection may therefore serve as a new diagnostic marker for postmenopausal osteoporosis.

Growing evidence shows that loss of estrogen leads to incomplete pluripotency genes expression and impairs stem cell self-renewal and multipotency, or may even trigger entry into an irreversible senescence state 23-25. However, strengthening these core TFs rejuvenates cellular function, and prevents cellular arrest state 26-28. In this study, we demonstrate that miR‑143/145, controlled by estrogen, directly targeted pluripotency genes NANOG, SOX2, and OCT4 transcripts and functionally repressed their expression. Additionally, SATB2, a key regulator of osteoblast differentiation 29, was also demonstrated as the targeted gene by miR-143/145, partially explaining the role of miR-143/145 in osteogenic differentiation. Previous studies reported that estrogen downregulated miR-145 expression 30 and several transcriptional repressors inhibited miR-143/145 promoter activity 31-33. Our results lend support to the molecular mechanism behind these findings, in that estrogen coupled with ERβ, but not with ERα, co-transcriptionally repressed miR-143 and miR-145 expression in mouse BMSCs. However, we could not identify an ERβ binding region on human miR‑143/145 promoters using bioinformatic analysis. Thus, the underlying regulatory mechanisms linking estrogen and miR-143/145 expression in human BMSCs require further investigation.

It is known that miRNAs are present in cytoplasm in the form of RISC with a core AGO2 component. Emerging research suggests that endogenous miRNAs could bind directly to promoters to regulate gene expression in response to different survival conditions 34-36. For example, miR-320 localized to the nuclei under hyperglycemic conditions 37 and miR‑210‑3p was highly expressed in nuclei after hypoxic stimulus 38. However, these studies didn't explain that how such miRNAs enter the nuclei, nor explain our findings that total miR‑143/145 levels were high in estrogen deficiency BMSCs while nuclear miR‑143/145 levels were low. Thus, we firstly put forward a regulatory mechanism that LncRNA MIR143HG acted in cytoplasm as a miR-143/145 sponge to protect pluripotency gene translation and, in nuclei, cooperates with miR-143 for binding to SOX2 promoter and activating its transcription by reducing H3K9me3 accumulation. Downregulation of MIR143HG under estrogen deficient conditions would therefore block core TFs translation and suppress SOX2 transcription. These double control mechanisms by MIR143HG and miR-143/145 strongly impact BMSCs function, and in turn, lead to imbalanced bone remolding under estrogen deficiency that contributes to postmenopausal osteoporosis. Furthermore, our findings and previous studies suggested that gene transcriptional activation was associated with H3K9me3 regulated by AGO2 20. Histone H3K9me3, a transcriptional silencing marker, is a key component of senescence-associated heterochromatin foci (SAHF), which is a hallmark of cellular senescence 39. Such miRNAs regulatory mechanisms would explain the increase in senescence identified here and in prior studies 40, 41.

Most drugs for the treatment of postmenopausal osteoporosis suppress bone resorption, which can lead to many long-term side effects such as osteonecrosis 42, 43. A key factor in the development of therapeutics for osteoporosis is to identify agents that not only block bone catabolism, but also enhance bone anabolism. Mounts of recent studies have uncovered the crucial role of osteoblast-dependent osteoclastic bone resorption by releasing EVs-encapsulated miRNA 44, 45. In this study, we uncovered that OVX BMSCs derived EVs transferred into osteoclast and significantly promoted osteoclastic differentiation which is attributed to carry high miR-143/145 levels. Moreover, miR-143/145 deletion mice significantly prevented OVX-induced bone loss by retaining osteoblastic bone formation and repressing osteoclastic bone resorption. Thus, the dual effect on BMSCs and osteoclasts function suggested miR-143/145 as proper targets to simultaneously regulate bone catabolism and anabolism. Nevertheless, it's undeniable that bone mass in knock out mice was lower than that in WT mice, probably because the regulation of miR-143/145 in vascular smooth muscle cells 17. Further, we explored the therapeutic and preventative value of small molecules by delivering chemically modified small RNAs of antagomiR-143/145 into bone marrow, which increased bone formation and decreased bone resorption, while similar experiments with agomiR-143/145 produced the opposite effects. Intriguingly, we noticed that mice injected with antagomir-143/145 exhibited decreased fat accumulation and increased blood vessel formation in bone marrow (Figure 7E), implying a potential role for miR-143/145 in adipogenesis and angiogenesis. Our data also reinforced the notion that circulating miR-143/145 might be sensitively diagnostic markers for postmenopausal bone loss since the starting of the miR-143/145 increase correlates well with the osteopenia onset which is around 50~55 years of age. However, whether they could serve as an early detection method to access the risk of postmenopausal osteoporosis require large samples investigation via prospective cohorts.

In conclusion, we put forward an extended regulatory mechanism in which miR-143/145 bidirectionally regulates core TFs expression involved the formation of miRNA-LncRNA complexes in cytoplasm and nucleus. Beyond the intra-BMSCs role, miR-143/145 are shuttled into osteoclasts by EVs and involved in osteoclastic differentiation, suggesting that targeting miR-143/145 has therapeutic potential for estrogen-deficient bone loss.

## Materials and Methods

### Mice

All experiments were performed with the approval of the Ethics Committee of the School of Stomatology of Nanjing Medical University. All procedures were carried out according to the guidelines of the Animal Care Committee of Nanjing Medical University. *miR-143/145^-/-^* mice were purchased from the Model Animal Research Center of Nanjing University (B6/N-miR-143/145^tm1^; Strain origin: C57BL/6N; Cat# T000090). The genomic sequence of miR-143/145 in mouse embryonic stem (ES) cells, was substituted with a DNA fragment containing an inverted phosphoglycerate kinase promoter-neomycin resistance cassette (PGK-Neo), which were flanked with loxP sites. Heterozygous miR-143/145 knock out mice were produced through deleting loxP-flanked PGK-neo cassette by Cre activity. We crossed heterozygous miR-143/145 to obtain homozygous mutants (*miR-143/145^-/-^* mice). In animal experiments, 3-month-old female WT mice and *miR-143/145^-/-^* mice were performed for ovariectomized operation to induce osteoporosis at least 6 mice per group. After two months, the mice were euthanized and the femurs and maxillae were harvested for micro-CT and histological analysis. Meanwhile, for tooth extraction operation, the first maxillary molar teeth were extracted based on our previous experience 46 and the maxillae were harvested after two weeks. Additionally, upon ovariectomized operation, OVX-induced WT mice were randomly divided into 3 groups, which were injected with antago/agomiR-143/145 (1 μM), antago/agomiR-NC (1 μM), and PBS (empty vector control) twice per month at a dose of 10 μl by periosteal injection into the marrow cavity of the femur 47. miRNA antagomiR or agomiR is specially labelled and chemically modified single-stranded small RNAs to directly penetrating the cell, which possessing high stability and can maintain activity for at least 2 weeks in bone marrow environments. Three months later, mice were euthanized and femur samples were harvested and processed for further analysis (n= 6 per group).

### Human subjects

The alveolar bone in female human mandible was harvested from volunteer donors who underwent dental implantation due to molar loss at least 1 year in affiliated hospital of stomatology of Nanjing Medical University. During the dental implantation surgery, we used a Ø 3.0 mm ring drill to obtain the alveolar bone from Premenopausal (25-35 years old) and Postmenopausal (65-75 years old) women. The serum and saliva were collected from 90 inpatients at our hospital who suffered from bone fracture, orthognathic, dental implantation or impacted tooth extraction surgery. Patients were received with a morning blood collection in the fasting state and the blood samples were centrifuged to get the serum samples for following assays. Additionally, at the same time, saliva samples were expectorated slowly into collection tubes with no stimulation from patients. For BMD measurement, according to latest guidelines for the diagnosis of osteoporosis in China with quantitative CT (BMD>120 mg/cm^3^, Normal; 80 mg/cm^3^<BMD<120 mg/cm^3^, Osteopenia; BMD<80 mg/cm^3^, Osteoporosis), the average BMD value of cancellous bone in two vertebras was analyzed by cone beam CT (CBCT). All subjects included in the study have written informed consent prior to samples collection through the approval of the Ethics Committee of the School of Stomatology of Nanjing Medical University. Inclusion criteria: (a) no jaw bone tumor or tumor-like lesion; (b) no intake of drugs affecting bone metabolism (corticosteroids, antiepileptic drugs, antituberculosis drugs, antacids containing aluminum, heparin); (c) no systemic disease that affected bone metabolism (hyperparathyroidism, hypoparathyroidism, osteochondrosis, renal osteodystrophy, deformans osteitis, osteogenesis imperfecta, diabetes).

### miRNA microarray

Total RNA was isolated from the Premenopausal and Postmenopausal human BMSCs and met the RNA quality control for array. The Oebiotech Company (Illumina, BGI, Shenzhen) performed the miRNA microarray assay. Briefly, miRNAs then detected by using Affymetrix GeneChip miRNA 4.0 arrays according to miRbase (www.mirbase.org). Biotin-labeled miRNAs were hybridized and processed for array and genechips were analyzed by Hewlett Packard Gene Array Scanner G3000 7G (Affymetrix). The data were obtained via Affymetrix Expression Console software and analyzed according to the MAS5 method.

### Cell culture and differentiation of BMSCs

Cells culture and differentiation were described in previous studies 40. Briefly, for human BMSCs isolation, the alveolar bone was wetted with complete medium (DMEM, 100 U/mL penicillin and 100 μg/mL streptomycin, 10% FBS) and cut into about 0.5mm pieces, and then adhered on culture plate. After 0.5 h, bone pieces were cultured with complete medium for 3 days. The media were replenished every three days and cells were passaged once BMSCs reached 80%-90% confluence. Three passages later, BMSCs were performed for the following assay.

For mice BMSCs isolation, bone marrow was flushed with α-MEM by an 18-gauge sterile needle inserted into the medullary cavity. The following protocols were in accordance with human BMSCs culture. To induce osteogenic differentiation, cells were cultured in an osteogenic medium containing complete medium supplemented with 50 μM ascorbic acid, 10 mM β-glycerophosphate and 10^-7^ M dexamethasone. Adipogenic induction was performed as follows: cells were cultured in complete medium containing 500 μM 3-isobutyl-1-methylxanthine (IBMX), 10 mg/l insulin, 1 μM dexamethasone and 0.2 mM indomethacin (Sigma, St. Louis, MO, USA). Both osteogenic and adipogenic differentiation media were replenished every three days.

For osteoclasts isolation, bone marrow were collected and cultured withα-MEM for 3 h to remove adherent cells. Then, non-adherent cells were harvested and re-seeded in on 96-well plates with complete medium with M-CSF (20 ng/mL) for the first 3 days. Then, these cells were refreshed with complete medium, M-CSF (20 ng/mL) and RANKL (20 ng/mL) every 3 days.

### Colony forming unit (CFU) assay and Cell senescence-associated β-galactosidase (SA-β-gal) staining

A total of 200 nucleated cells were seeded in six-well plates in triplicate. To establish colonies, BMSCs were cultured in complete medium for 10 days without changing medium. Then, cell colonies were fixed in 4% paraformaldehyde and stained with a crystal violet solution (Sigma, St. Louis, Mo). After staining, aggregates >50 positive cells were counted as colonies.

BMSCs at 5 passages were seeded in 12-well plates and incubated with staining solution according to SA-β-gal kit. After 16 h co-culture, Five randomly selected fields were chosen for quantification of SA β-gal positive cells under inverted microscopy.

### Alizarin red staining, Von Kossa staining and Oil red O staining

BMSCs were cultured in osteogenic induction for 10-14 days *in vitro*, and then stained using alizarin red at room temperature for 10 min. The deposition of calcium was identified under a light microscope. Calcified nodules were destained with 10% cetylpyridinium chloride, and the absorbance of calcium concentration was measured at 450 nm and quantified after controlling to cell numbers of each group. Additionally, calcified nodules were stained with 2% silver nitrate solution under the ultraviolet light exposure until calcified deposition were turned black. After 14 days adipogenic induction, the lipid droplets were detected by Oil Red O staining. The proportion of positively stained cells in the total area was measured using ImageJ software. Five random fields from each sample were captured under a light microscope and chosen for quantification.

### Trap staining, F-actin staining and Bone resorption

Mature osteoclasts were fixed with 4% paraformaldehyde and stained by tartrate-resistant acid phosphatase (TRAP) kit. More than two positively stained nuclei were identified as matured BMSCs. Additionally, for F-actin ring staining, the fixed cells were incubated with 5 μg/mL Phalloidin for half an hour. Non-adherent cells were cultured on bovine bone slides to differentiate into osteoclasts. After 12 days, bone resorption pits were washed with PBS, stained with 0.01 g/l toluidine blue to indicate resorption pits and visualized with the Leica Application Suite (Leica, Mannheim, Germany).

### Immunofluorescence

BMSCs were seeded on coverslips for 48 h at 37 °C, fixed with 4% paraformaldehyde and permeabilized with 1% Triton X-100. After pre-incubated with goat serum to block nonspecific staining, primary antibodies were incubated with cells overnight at 4 °C. then, coverslips were rinsed with TBS and incubated with FITC or Cy3-labeled secondary IgG at 37 °C for 1 h. Coverslips were labeled with DAPI at room temperature for 1.5 min and images were captured under fluorescence microscope in five random chosen fields from three independent samples (Leica Microsystems, Mannheim, Germany).

### Fluorescent *in situ* hybridization (FISH)

All FISH probes were designed and synthesized by Genepharma (Shanghai, China). MIR143HG, miR-143, miR-145 subcellular localization in BMSCs were examined by FISH kit (Genepharma, Shanghai, China). Briefly, BMSCs were seeded on coverslips, fixed with 4% paraformaldehyde for 15 min at 4 °C, and permeabilized with 1% Triton X-100 for 30 min. then, the coverslips were washed with 2 × SSC for 30 min at 37 °C and dehydrated by ethanol at room temperature. FISH probes (miR-143, 5ʹ- GAGCTACAGTGCTTCATCTCA-3ʹ; miR-145, 5ʹ-AGGGATTCCTGGGAAAACTGGAC-3ʹ; MIR143HG, 5ʹ-AGAGATGCAGCACTGCACCTCA-3ʹ) in hybridization buffer were incubated with coverslips overnight at 37 °C. Subsequently, coverslips were washed twice with 0.4×SSC/0.3%Tween20 and 2×SSC/0.1%Tween20, stained with DAPI for 1.5 min, and then detected by confocal fluorescence microscope.

### RNA-Binding Protein Immunoprecipitation (RIP) Assay

RIP assay was designed according to MS2bp-MS2bs system according to a previous study 48. Briefly, 293T cells were seeded in cultural plates for 24 h, and then co-transfected with 30 μg pcDNA3.1-MS2bs plasmid including Nanog, SOX2, OCT4, SATB2, Renilla luciferase (as blank control vectors), 10 μg MS2bp-GFP plasmid and miR143/145 mimics by Lipofectamine 2000 (Invitrogen). After 48 h, cells were detected for RNA immunoprecipitation using GFP antibody based on EZ-Magna RIP^TM^ Kit (Millipore). The complexes of purified RNA were performed for RT-PCR and co-isolated RNA-binding proteins were detected by Western blot.

### Luciferase assays

293T cells were seeded in 24-well plates for 24 h, and then were were co-transfected with pGL3-basic luciferase reporter vector, Renilla vector (pRL-TK; Promega, Madision, WI, USA), miRNA control and miR-143/145 mimics or miR-143/145 inhibitors using Lipofectamine 2000 (Invitrogen). Luciferase activities were measured 48h after transfection by using the Dual-Luciferase Reporter Assay System (Promega, Madision, WI, USA). Firefly luciferase activity was normalized to Renilla luciferase activity for each sample.

### Chromatin immunoprecipitation (ChIP) assays

In this study, based on CUT&Tag techniques 49, ChIP assays was performed according to hyperactive in-Situ ChIP kit (Vazyme, Nanjing, China). Briefly, after collected and rinsed with wash buffer, cells were resuspended with concanavalin A coated magnetic beads (ConA beads) in PCR tube. The binding cells to beads were incubated with primary antibody overnight at 4 °C. Then, the tubes were placed on magnetic separator and pulling off the supernatant. The beads were co-cultured with Dig-wash Buffer containing secondary antibody (Bioworld) for 1 h. Beads were washed with dig-wash buffer using magnetic separator to removing unbound antibodies. Hyperactive pG-Tn5/pA-Tn5 Transposon were diluted 1:50 in Dig-300 Buffer and added into tubes for 1 h with gentle vortexing. Next, the beads were washed with Dig-300 Buffer and resuspended in 300 μl tagmentation buffer to generate DNA fragments. The tagmentation was stop by a mixed liquid containing 10 μl 0.5M EDTA,3 μl 10% SDS and 2.5 μl 20 mg/mL Proteinase K, and subsequently, the DNA were eluted by chloroform, ethanol and Tris-HCl. The purified DNA was ready for RT-PCR enrichment.

### Isolation of EVs derived from BMSCs

To collect the BMSCs derived EVs, cells reaching approximately 80-90% confluence were washed with PBS three times and cultured in serum-free αMEM for 48 h. Then, the supernatant was harvested and centrifuged at 300×g for 10 min to remove cell lysate, 2000×g for 15 min to remove cell debris and 10,000×g for 30 min to remove large EVs and collecting the supernatant each time. Subsequently, the supernatant was centrifuged at 100,000×g for 70 min twice to remove the contaminating protein, and the pellet was collected each time and resuspended with PBS. EVs that mainly contained microvesicles and exosomes in the pellet were resuspended with 100 µL PBS and stored at -80 °C.

### Western blot

Western blot was performed according to previous studies. We lysed the cells with RIPA buffer (Beyotime, Shanghai, China) containing 10 mM protease inhibitor (PMSF; Beyotime). Protein lysate was loaded onto 10-15% SDS-PAGE gels and then transferred to PVDF membranes (Millipore, Billerica, MA, USA). Prior to overnight incubation with primary antibodies (Table S2), membranes were blocked by 5% fat-free milk for 2 h. Detailed information regarding the primary antibodies is listed in Table S2. After washing with TBST three times, the membranes were incubated for 1 h with the corresponding horseradish peroxidase-conjugated secondary antibodies (1:10,000). We observed the blots using an ECL detection kit (Millipore) and examined the Western blotting results by ImageJ software. In each group, the relative protein levels were quantified as the ratio of the level of the protein of interest to the level of β-actin.

### Real-time RT-PCR

Total RNA was extracted from cells using Trizol reagent according to the manufacturer's instructions. Reverse transcription was performed with 1 μg of total RNA in a final volume of 20 μL using the PrimeScript RT reagent kit (Takara Bio, Shiga, Japan) according to the manufacturer's recommendations. The levels of each mRNA or miRNA were normalized to the GAPDH or U6 levels, respectively.

miR-143/145 in EVs from human samples or mice BMSCs supernatant were extracted using the miRNeasy Serum/Plasma kit (QIAGEN, Valencia, CA, USA) according to the manufacturer's instructions. miRNA in EVs were purified and detected by the All-in-One miRNA qRT-PCR Detection Kit (GeneCopoeia, Rockville, MD, USA). To normalize miRNA expression, miR-143/145 levels were compared to spiked-in ce-miR-39, which was applied as the reference using the miRNeasy Serum/Plasma Spike-In Control kit (QIAGEN, Valencia, CA, USA). Each experiment was performed in triplicate. The primer sequences used in this study are listed in Table S1. The 2^-ΔΔCT^ method was used to quantify expression of the genes of interest.

### Micro computed tomography (micro-CT) analysis and Histological observation

The micro-architectural properties of the distal femur and tooth extraction socket were analysed using the micro-CT system (Skyscan 1176, Kontich, Belgium). Bones were scanned at a high resolution (18 μm) with an energy of 50 kV and 456 μA. We applied NRecon v1.6 and CTAn v1.13.8.1 software to reconstruct and analyse the 3D images of the bone. The region of interest (ROI) was defined as the area proximal to the growth plate in the distal femurs. To evaluate the trabecular bone structure, the following five parameters were calculated: the bone volume ratio (BV/TV, %), trabecular thickness (Tb.Th.), trabecular number (Tb.N.), trabecular separation (Tb.Sp.) and bone mineral density (BMD). The region of mid-diaphysis on the 10% femoral length was selected to analyze the cortical thickness (Ct. Th).

Bone tissue was fixed with 4% paraformaldehyde for 48 h, decalcified in 10% ethylene diaminetetraacetic acid (EDTA), embedded in paraffin wax, sectioned into 4-μm-thick slices and stained as previously described. Briefly, bone structure was observed by HE and masson trichrome staining. The quantification of osteoblasts was presented as graphs of quantification of Ob. S/BS (osteoblast surface per bone surface) and Ob. N/BS (osteoblast number per bone surface), as well as Tb. N OCN^+^/BS (OCN positive bone cell number per bone surface) by IHC. Osteoclasts were observed by TRAP staining and quantified by Oc. N/BS (osteoclast number per bone surface) and Oc. S/BS (osteoclast number per bone surface).

### Fluorescence labeling analysis

To observe the mineralizing front, the mice were given a subcutaneous injection with 20 mg/kg calcein (Sigma, USA) and 30 mg/kg alizarin red S (Sigma, USA) at 8 and 2 days respectively before euthanasia. The undecalcified bone samples were dehydrated in 75 to 100% ethanol and embedded in pure resin blocks after being soaked in diluted resins. The blocks were cut along the centre of the femur axis to obtain 150-mm-thin sections (EXAKT, Germany) and were further thinned between 15 and 20 by abrasive paper. The pictures were captured by confocal microscopy to study the new bone formation. The histomorphometric analysis of MAR and BFR/BS were quantified by Image pro software to analyze the bone dynamics. Additionally, the sections were stained with 2% silver nitrate solution under the ultraviolet light exposure until calcified deposition were turned black. Then, the sections were rinsed with 5% sodium thiosulfate and captured by a light microscope.

### Statistical Analysis

The results are expressed as the means ± SD. Experiments were repeated independently at least three times. Statistical significance of two groups comparison was assessed using Student's t-test. Analysis across multiple comparisons were performed for one-way ANOVA. To determine significance between WT and *miR-143/145^-/-^*mice in response to Sham and OVX operation, comparisons were made using two-way ANOVA. *p* < 0.05 (*), *p* < 0.01 (**) and *p* < 0.001 (***) was considered statistically significant.

## Supplementary Material

Supplementary figures and tables.
