# Supplementary material for: Identification of the canonical and noncanonical role of miR-143/145 in estrogen-deficient bone loss
Source: Theranostics. 2021 Mar 13;11(11):5491–510. doi: 10.7150/thno.55041 (PMC8039936; doi:10.7150/thno.55041)
Supplement: Supplementary file 1 — Supplementary figures and tables. [file thnov11p5491s1.pdf]

# Supplemental Information

## Supplemental Figures

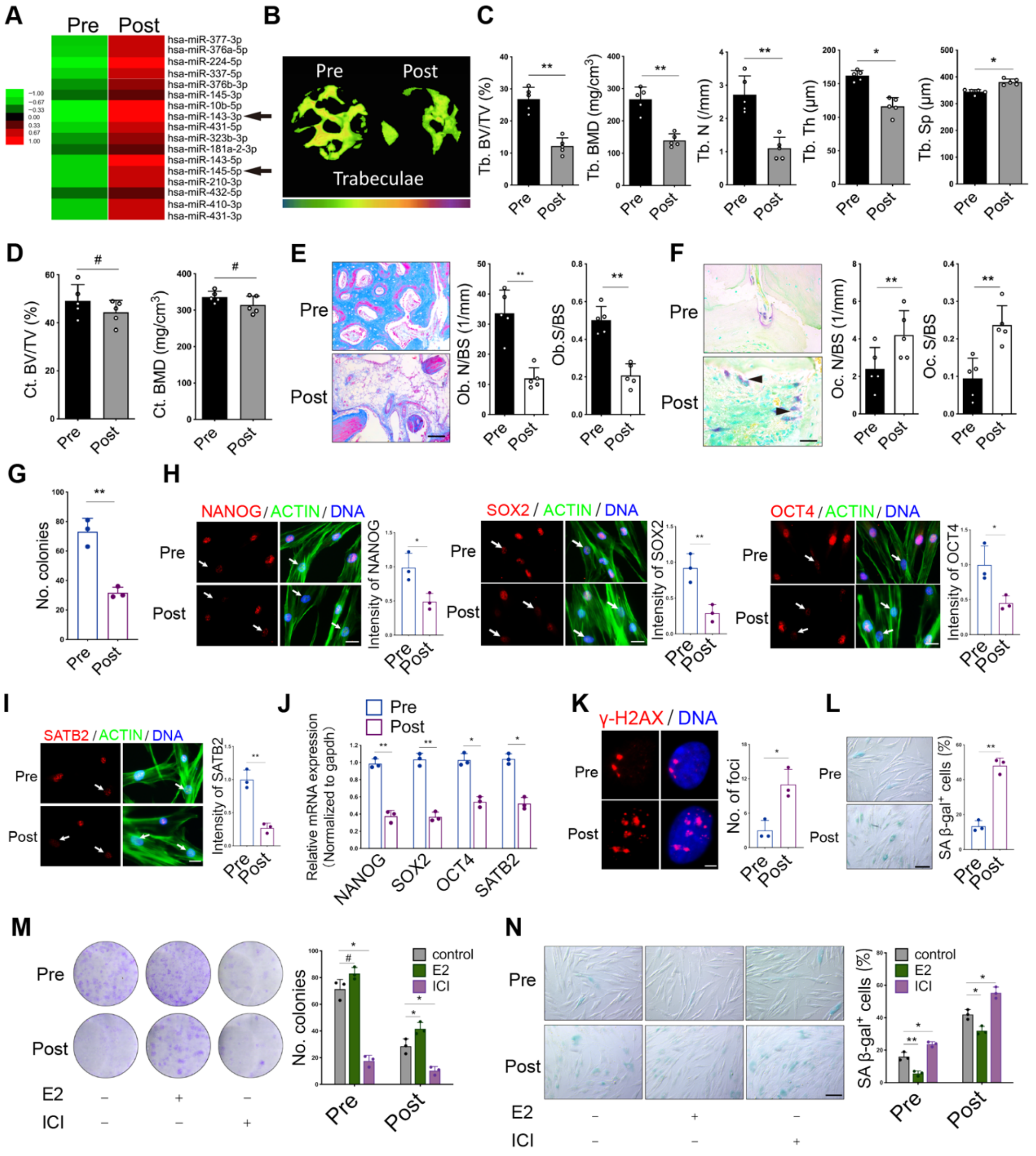

**Fig. 1. Increases in miR-143/145 levels during estrogen-deficient osteoporosis and the attenuation of BMSC function**

**A** Heatmap analysis of the miRNAs profiling in Pre- and Post-BMSCs. **B** The trabecular thickness in cancellous bone was color coded, which blue-green color indicates thinner trabeculae and yellow-red color indicates thicker trabeculae. **C** Quantitative measurements of Tb. BV/TV, Tb. BMD Tb. N, Tb. Sp, and Tb. Th of trabeculae were calculated. **D** Cortical bone from dental implantation surgery was quantified for Ct. BV/TV and BMD analysis. **E** Representative masson trichrome

staining images and quantitative for Ob.N/BS and Ob.S/BS analysis were showed. Scale bars: 200 μm. **F** Representative TRAP staining images and quantification of N.Oc/BS (osteoclast number / bone surface). Arrow head indicated the positive staining osteoclasts. Scale bars: 50 μm. **G** The quantification of colonies was analyzed in Pre- and Post-BMSCs. **H, I** Immunostaining of NANOG, SOX2, OCT4 and SATB2 expression in BMSCs. White arrows indicated the BMSCs with decreased core TFs and the mean intensity was measured with ImageJ. Scale bars: 50 μm. **J** qRT-PCR showing NANOG, SOX2, OCT4 and SATB2 mRNA expression in Pre- and Post-BMSCs. **K** γH2AX foci and **(L)** SA-β-gal staining was examined to indicate the senescence difference. Right panel showed the analysis of γH2AX foci numbers and SA-β-gal positive cells. Scale bars: 4 μm **(K)**; 100 μm **(L)**. **M** CFU assays showed that estrogen potentiated colony numbers and ICI reduced the colonies in Pre- or Post-BMSCs. **N** BMSCs stimulated with estrogen or ICI were analyzed for senescence state by SA-β-gal staining. Scale bars: 100 μm. Results are presented as the mean ± S.D. $^{*}p < 0.05$; $^{**}p < 0.01$; $\#p > 0.05$ by Student's t test and one-way ANOVA.

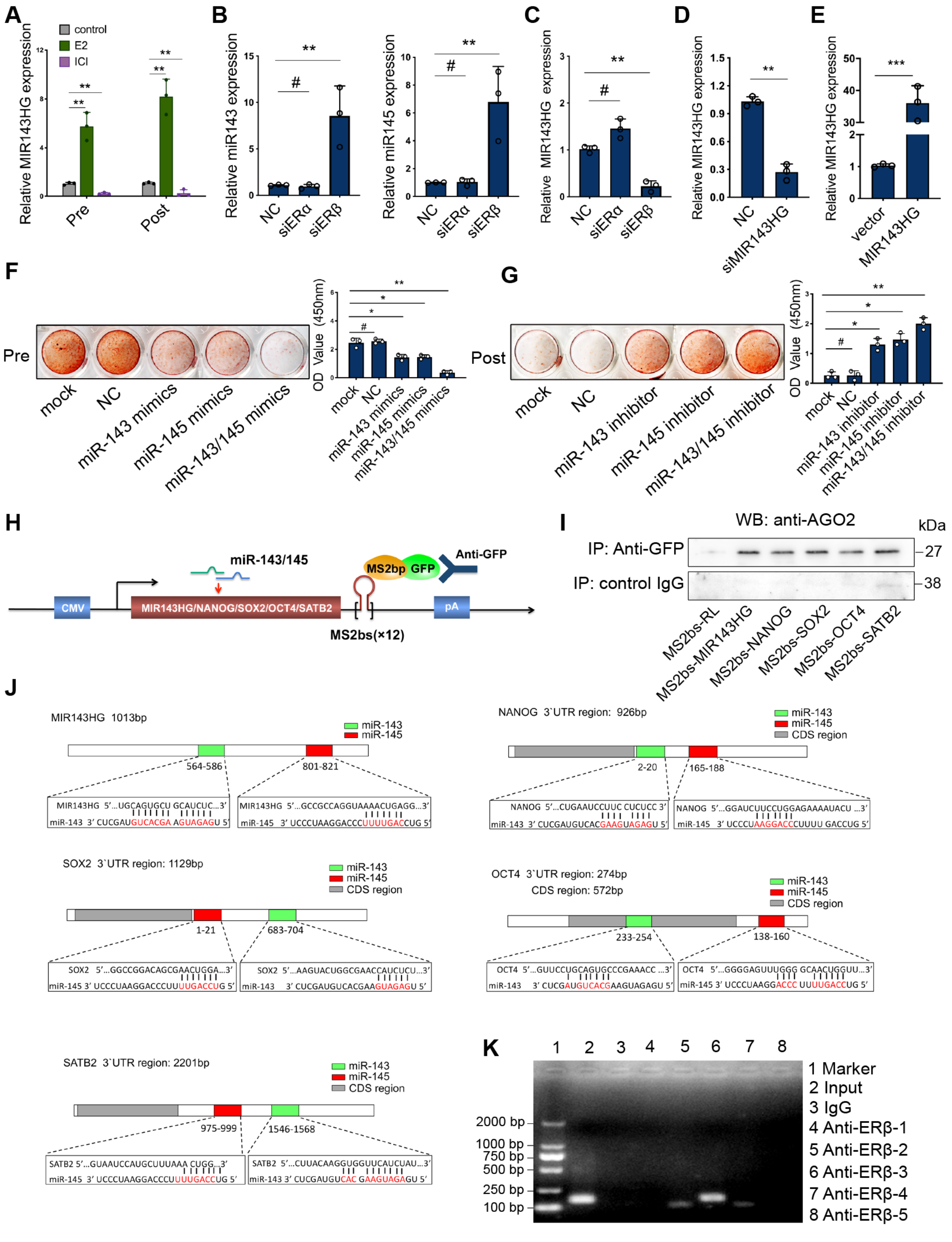

**Fig. 2. ERβ activates MIR143HG transcription to regulate the function of miR-143/145 that target core TFs and SATB2**

**A** MIR143HG was detected in estrogen or ICI treated BMSCs. **B, C** qRT-PCR showing the miR-143/145 and MIR143HG expression in siERα or siERβ transfected BMSCs. **D, E** Analysis for knockdown efficiency of MIR143HG siRNA (siMIR143HG) and enhancement efficiency of MIR143HG overexpressing plasmid. **F** Observation and quantitation of calcified nodules formation in Pre BMSCs that transfected with miR-

143/145 mimics by alizarin red staining. **G** Calcified nodules formation and quantitation in Post BMSCs. **H** MS2 system was applied for RIP assay, in which MS2bp specifically binding MS2bs. **I** After precipitated by GFP antibody or IgG, 10% volume of RNA-protein complexes were extracted and subjected to Western blot assay with AGO2 antibody. **J** The putative binding sites of miR-143/145 on MIR143HG, NANOG, SOX2, OCT4 and SATB2 transcripts were predicted by targetscan or Miranda. The red nucleotides indicated the putative paired sites. **K** The amplification products of DNA fragment from ChIP assay were then performed for gel shift assay. E2, estrogen. Results are presented as the mean ± S.D. *$p < 0.05$; **$p < 0.01$; ***$p < 0.001$; #$p > 0.05$ by Student's t test and one-way ANOVA.

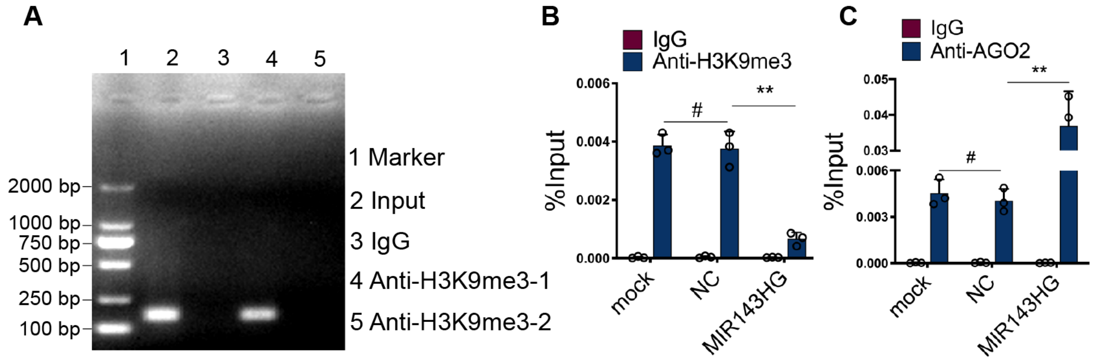


**Fig. 3. MIR143HG guides miR-143/145 into nuclear and cooperatively regulates SOX2 transcription**

**A** Amplified DNA using prime 1 or prime 2 were further examined by gel shift assay. **B, C** BMSCs transfected with mock, NC or MIR143HG overexpressing vector were subjected to ChIP assay by H3K9me3 or AGO2 antibody. Results are presented as the mean ± S.D. $^{**}p < 0.01$; $^{\#}p > 0.05$ by Student's t test and one-way ANOVA.

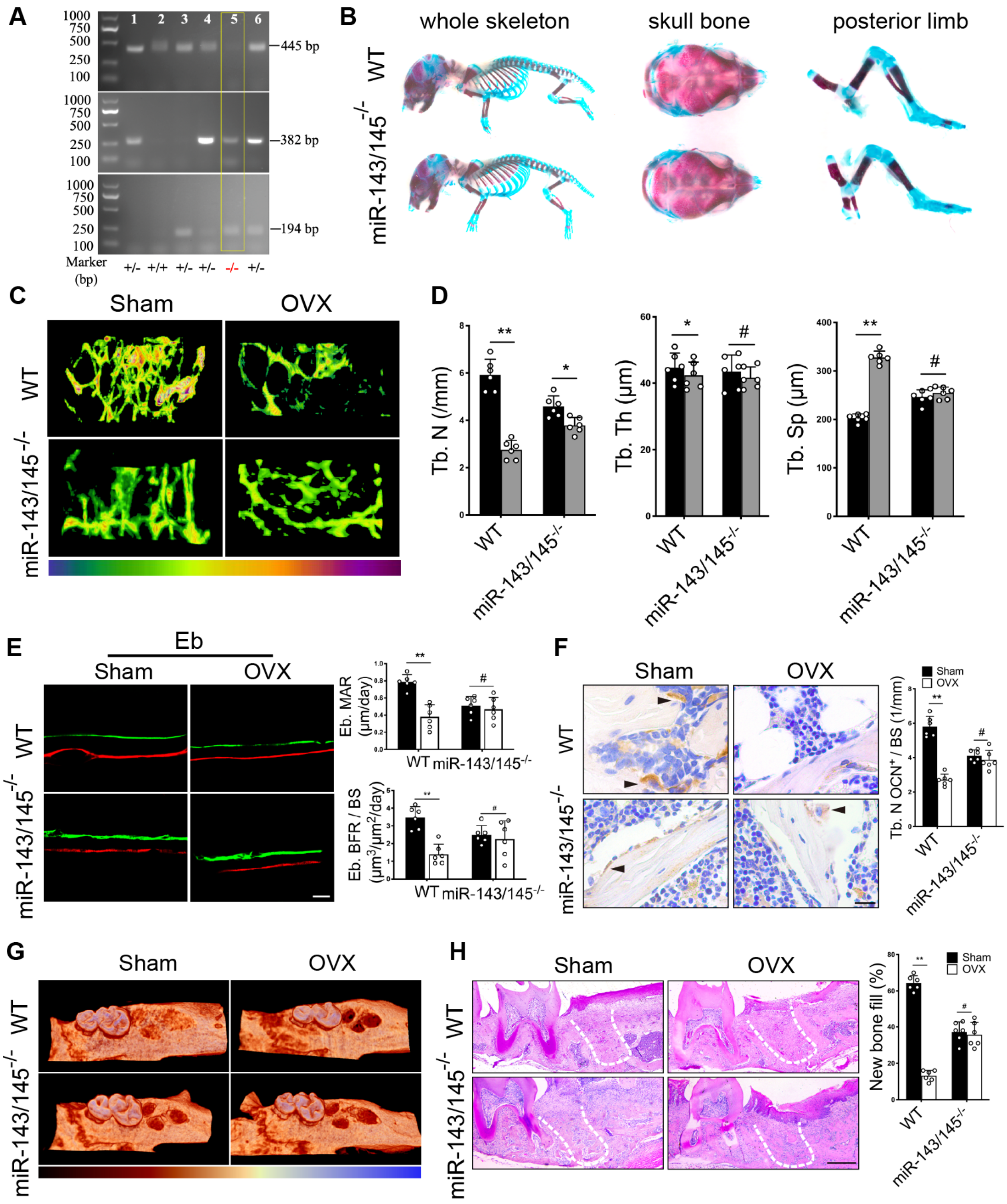


**Fig. 4. Depletion of miR-143/145 in mice prevented bone loss and potentiated bone regeneration in OVX-induced osteoporosis**

**A** All mice were genotyped by PCR from tail snip DNA**. B** Whole skeleton staining revealing the whole mount skeleton, skull bone, posterior limb using Alizarin red and Alcian blue. **C** Trabecular thickness maps were presented, which blue-green color indicates thinner trabeculae and yellow-red color indicates thicker trabeculae. **D** Quantitative measurements of Tb. N, Tb. Sp, Tb. Th. **E** Representative images of dynamic histomorphometry of endosteal (Eb) bone with quantification of mineralization apposition rate (MAR) and bone formation rate per bone surface (BFR/BS). Scale bars: 20 μm. **F** Representative OCN positive osteoblasts and

quantification on the trabecular by IHC. Scale bars: 50 μm. **G** Micro-CT analysis of new bone fill in tooth extraction socket by color coded thickness maps. Color changes from red to blue denote a gradual elevation in bone thickness. **H** Representative HE staining showing the new bone mass and quantification of new bone fill in tooth extraction socket, which was delineated by white dotted lines. Scale bars: 100 μm. Results are presented as the mean ± S.D. *$p < 0.05$; **$p < 0.01$; ***$p < 0.001$; #$p > 0.05$ by two-way ANOVA, n = 6 mice per group.

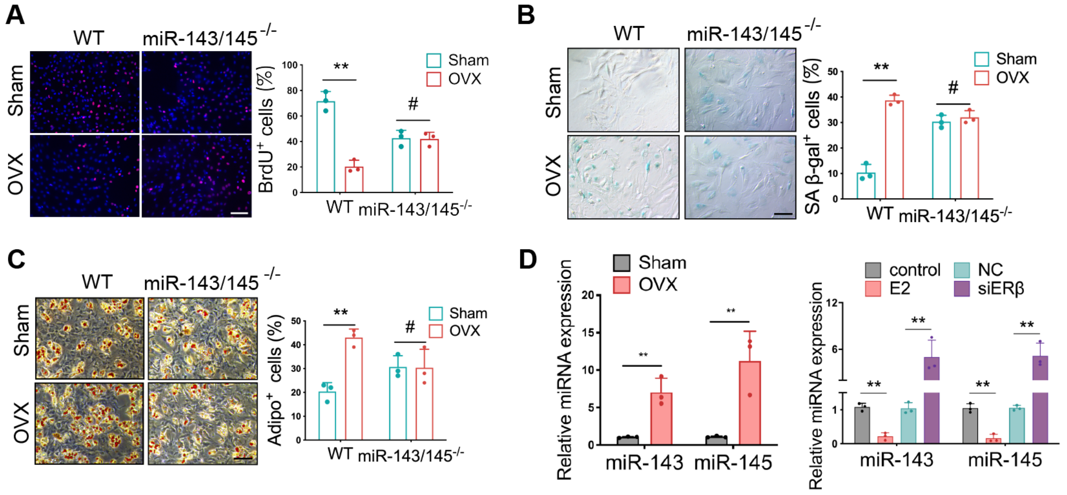


**Fig. 5. miR-143/145 depletion counteracts the adverse effects of estrogen deficiency on BMSCs function**

**A** BrdU assay revealing proliferation ability in Sham and OVX-induced WT and *miR-143/145*$^{-/-}$ BMSCs. Scale bars: 100 μm. **B** SA-β-gal showing the senescence difference in WT and *miR-143/145*$^{-/-}$ BMSCs following OVX. Right panel shows the respective quantification. Scale bars: 100 μm. **C** Oil red O staining showing the ability of adipogenic differentiation. Right panel shows the respective quantification. Scale bars: 100 μm. **D** Detection of miR-143/145 in OVX BMSCs and estrogen or siERβ treated BMSCs. Results are presented as the mean ± S.D. *$p < 0.05$; **$p < 0.01$ by Student's t test and one-way ANOVA.

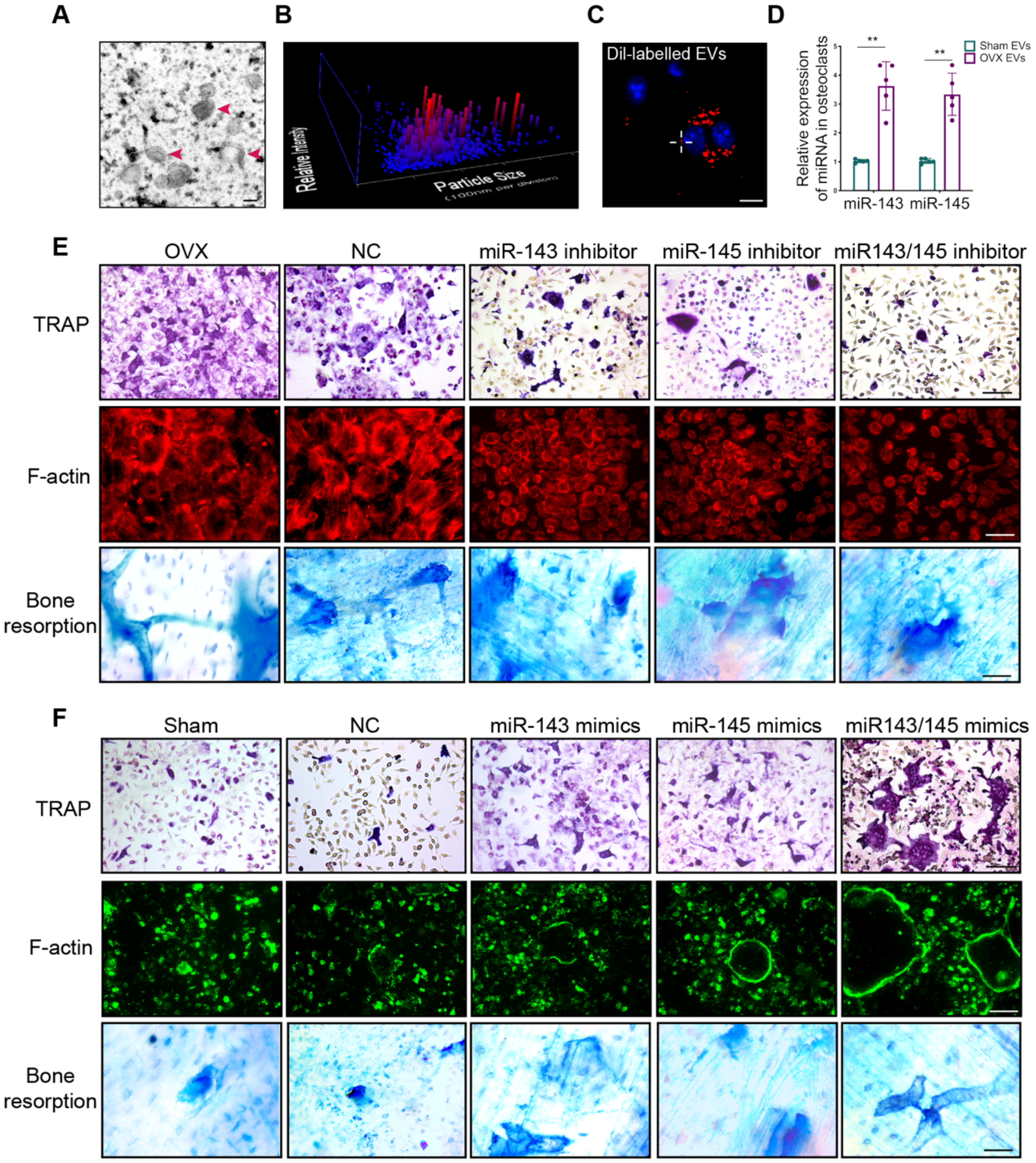


**Fig. 6. EVs loaded miR-143/145 from BMSCs activates osteoclast function**

**A** An overview TEM image of EVs that indicated by red arrows were captured to understand the whole EVs population. Scale bars: 100 nm. **B** The 3D graph (particle size vs. Relative intensity vs. particle number concentration) of NTA for calculating EVs size distribution. **C** Fluorescent microscopy analysis revealing the DiI-labeled EVs incorporated into osteoclasts. Scale bars: 50 µm **(C)**. **D** Osteoclasts co-cultured with EVs from Sham or OVX-induced BMSCs were examined by qRT-PCR. **E, F** Representative images of TRAP positive staining, F-actin ring staining, and bone resorption area in miR-143/145 inhibitor or mimics treated osteoclasts. Scale bars: 100 µm **(E, F)**. Results are presented as the mean ± S.D. *$p < 0.05$; **$p < 0.01$ by Student's t test.

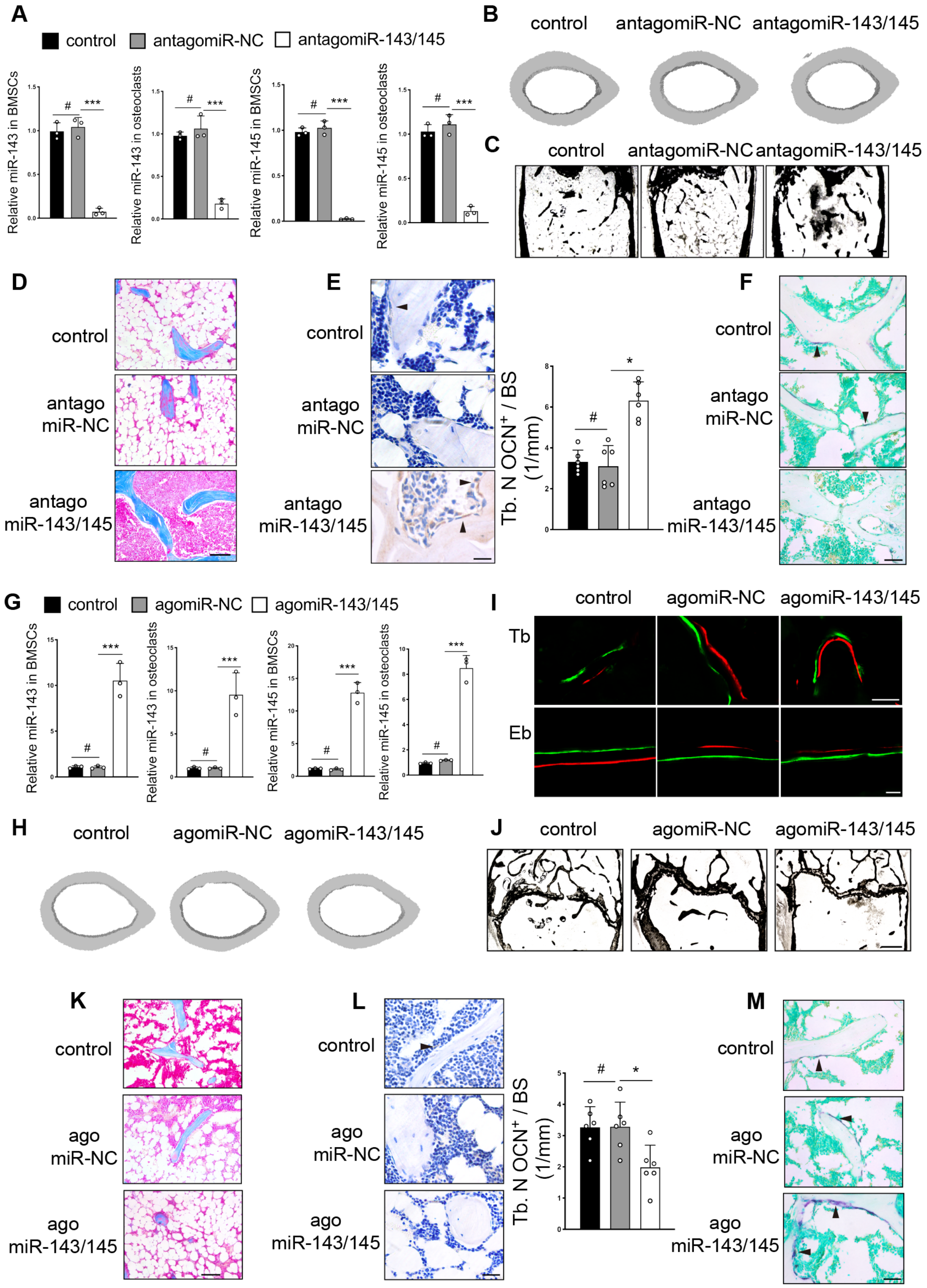


**Fig. 7. AntagomiR-143/145 prevents and agomiR-143/145 exacerbates OVX-induced bone loss**

**A** qRT-PCR analysis of miR-143/145 expression in BMSCs and osteoclasts of mice with antagomiR. **B** Representative micro-CT images of cortical bone from control, antagomiR-NC, and antagomiR-143/145 treated femurs. **C** Von Kossa staining showing

an overall trabecular bone mass. Scale bars: 500 μm. **D** Masson trichrome staining revealing the trabecular bone accounts. Scale bars: 100 μm. **E** IHC showing the OCN positive osteoblasts. Arrow head indicated the positive staining osteoblasts. Scale bars: 50 μm. **F** TRAP staining showing osteoclastic activity. Arrow head indicated the positive staining osteoclasts. Scale bars: 50 μm. **G** qRT-PCR analysis of miR-143/145 expression in BMSCs and osteoclasts with agomiR. **H** Micro-CT images of cortical bone from control, agomiR-NC, and agomiR-143/145 treated mice. **I** Representative images of dynamic histomorphometry of Tb and Eb bone. Scale bars: 20 μm. **J** Representative images of von Kossa showing an overall trabecular bone mass. Scale bars: 500 μm. **K** Representative images of masson trichrome staining revealing the trabecular bone mass and quantification of osteoblast numbers and surface on the trabecular, **(L)** as well as OCN positive osteoblasts by IHC. Scale bars: 100 μm **(K)**; 50 μm **(L)**. Arrow head indicated the positive staining osteoblasts. **M** Representative images of TRAP staining showing osteoclastic activity. Arrow head indicated the positive staining osteoclasts. Scale bars: 50 μm. Results are presented as the mean ± S.D. *$p < 0.05$; #$p > 0.05$, by one-way ANOVA, n = 6 mice per group.

**Supplemental Table**

**Table S1. Primer sequence for qRT-PCR and ChIP qPCR.**

| Gene | Primer Sequence |
|---|---|
| Primer for human GAPDH | F 5′-GGAGATTACTGCCCTGGCTCCTA-3′ |
| | R 5′-GACTCATCGTACTCCTGCTTGCTG-3′ |
| Primer for human NANOG | F 5′-AAGGTCCCGGTCAAGAAACAG-3′ |
| | R 5′-CTTCTGCGTCACACCATTGC-3′ |
| Primer for human SOX2 | F 5′-GGCAGAGAAGAGAGTGTTTGC-3′ |
| | R 5′-GCCGCCGATGATTGTTATT-3′ |
| Primer for human OCT4 | F 5′-TGAGAGGCAACCTGGAGAAT-3′ |
| | R 5′-AACCACACTCGGACCACATC-3′ |
| Primer for human SATB2 | F 5′-CCAGGAGTTTGGGAGATGGTAT-3′ |
| | R 5′-GTGAGGAGACTGTTCGTTGGTT-3′ |
| Primer for human MIR143HG | F 5′-AGAGCCGCCAGGTAAAAC-3′ |
| | R 5′-TCCAACCCCACCAAAGG-3′ |
| Primer for mouse pri-miR-143 | F5′-GTCTCCAGGGCGTGTCCAGACCAGTA-[illegible] |
| | R 5′ CCTGAGCTACAGTGCTTCATCTCAGA-3′ |
| Primer for mouse pri-miR-145 | F 5′-GTCCAGTTTTCCCAGGAATCCCTT-3′ |
| | R 5′ GTCCCAAGACCGCTTACCTCCCTC-3′ |
| Primer for human MIR143HG Promotor-1 (ChIP-ERβ) | F 5′-TGAGTCATAGCTTACATC-3′ |
| | R 5′-GGAACTAACTTCTGTGAA-3′ |
| | F 5′-CCACTCAGGATGTCACAAG-3′ |

| | |
|---|---|
| Primer for human MIR143HG Promotor-2 (ChIP-ERβ) | R 5′-CTCATTCACAGTTCCTCAGA-3′ |
| Primer for human MIR143HG Promotor-3 (ChIP-ERβ) | F 5′-CTCTGGGAAGAAGGCATTT-3′ |
| | R 5′-GGGTTTGAATGTGGAATGTC-3′ |
| Primer for human MIR143HG Promotor-4 (ChIP-ERβ) | F 5′-CCTGAAGGACATTCCACAT-3′ |
| | R 5′-GAAGCTACTCCCATCATCAT-3′ |
| Primer for human MIR143HG Promotor-5 (ChIP-ERβ) | F 5′-TCTCAGGCTTAATGACTTC-3′ |
| | R 5′-AAGGAGTGGTTGACAATT-3′ |
| Primer for human SOX2 Promotor-1 (ChIP-RNAa complex) | F 5′-AAGGTTAGTAAGGAACAA-3′ |
| | R 5′-TTCTTCTGTAACACTCTC-3′ |
| Primer for human SOX2 Promotor-2 (ChIP-RNAa complex) | F 5′-TGTAGCGACAACAAGAGAA-3′ |
| | R 5′-GTTAGAGGAGGATGAGATGG-3′ |
| Primer for mouse miR-143/145 Promotor (ChIP-ERβ) | F 5′-AGGGACCCCAAATCATAA-3′ |
| | R 5′-AACCTACCTCATCCTCTG-3′ |

**Table S2. Information of Antibodies, Chemicals Reagent, Commercial Kit, Cell Lines, mouse Strains, Recombinant DNA and Software in this study.**

| REAGENT or RESOURCE | SOURCE | Cat# |
|---|---|---|
| **Antibodies (Dilution)** | | |
| Rabbit polyclonal anti-NANOG (1:500) | Santa Cruz | Cat# sc-33760 |
| Rabbit monoclonal anti-SOX2 (1:1000) | Abcam | Cat# ab93689 |
| Rabbit monoclonal anti-OCT4 (1:1000) | Abcam | Cat# ab181557 |
| Rabbit polyclonal anti-SATB2 (1:1000) | Abcam | Cat# ab69995 |
| Mouse monoclonal anti-BrdU (1:500) | Proteintech | Cat# MS-396-P1 |
| Mouse monoclonal anti-γH2AX (1:1000) | Abcam | Cat# ab26350 |
| Rabbit polyclonal anti-P53 (1:800) | Proteintech | Cat# 10442-1-AP |
| Rabbit polyclonal anti-P21 (1:500) | Proteintech | Cat# 10355-1-AP |
| Rabbit polyclonal anti-GFP (1:1000) | Abcam | Cat# ab290 |
| Rabbit monoclonal anti-AGO2 (1:1000) | Cell Signaling Technology | Cat# 2897 |
| Rabbit polyclonal anti-H3K9me3 (1:1000) | Abcam | Cat# ab8898 |
| Mouse monoclonal anti-LMNA (1:1000) | Cell Signaling Technology | Cat# 86846 |
| Rabbit polyclonal anti- RUNX2 (1:1000) | Cell Signaling Technology | Cat# AP12556PU-N |
| Rabbit polyclonal anti-OCN (1:1000) | Abcam | Cat# ab93876 |
| Rabbit polyclonal anti-CD226 (1:500) | Affinity Bioscience | Cat# AF0087 |
| Rabbit Polyclonal anti-SRGAP2 (1:500) | Proteintech | Cat# 22519-1-AP |
| Mouse monoclonal anti-CD63 (1:1000) | Abcam | Cat# ab213090 |
| Rabbit monoclonal anti-CD9 (1:1000) | Abcam | Cat# ab92726 |
| Rabbit Polyclonal anti-Estrogen Receptor beta (1:1000) | Abcam | Cat# ab3577 |

| **Chemicals, Peptides, and Recombinant Proteins** | | |
|---|---|---|
| DMEM, low glucose | Thermo Fisher Scientific | Cat# 11885084 |
| MEM Alpha basic | Thermo Fisher Scientific | Cat# 12571063 |
| Fetal Bovine Serum | Thermo Fisher Scientific | Cat# 16140071 |
| Penicillin-Streptomycin | Sigma-Aldrich | Cat# V900929 |
| Red Blood Cell Lysis Buffer | Beyotime | Cat# C3702 |
| Estrogen | Sigma-Aldrich | Cat# 1250008 |
| Estrogen Receptor Antagonist, ICI 182,780 | Sigma-Aldrich | Cat# 5. 31042 |
| L-Ascorbic acid | Sigma-Aldrich | Cat# A5960 |
| β-Glycerophosphate | Sigma-Aldrich | Cat# E2758 |
| dexamethasone | Sigma-Aldrich | Cat# D4902 |
| Insulins | Sigma-Aldrich | Cat# I0908 |
| 3-isobutyl-1-methylxanthine | Sigma-Aldrich | Cat# I5879 |
| Indomethacin | Sigma-Aldrich | Cat# I7378 |
| Oil Red O | Sigma-Aldrich | Cat# O0625 |
| Anti-Murine M-CSF | PeproTech | Cat# 500-P62G |
| Recombinant Murine sRANK Ligand | PeproTech | Cat# 315-11 |
| Lipofectamine™ 2000 Transfection Reagent | Invitrogen | Cat# 11668027 |
| TRIzol™ LS Reagent | Invitrogen | Cat# 10296010 |
| Calcein | Sigma-Aldrich | Cat# C0875 |
| Alizarin Red S | Sigma-Aldrich | Cat# A5533 |
| DiIC18(3) ( Ex/Em:550/567nm) | YEASEN | Cat# 40726ES10 |

| | | |
|---|---|---|
| **Critical Commercial Assays** | | |
| miRNeasy Serum/Plasma kit | QIAGEN | Cat# 217184 |
| TRAP staining kit | Sigma-Aldrich | Cat# N2250 |
| β-Gal Staining Kit | GenMed | Cat# GMS10012.1 |
| All-in-One miRNA qRT-PCR Detection Kit | GeneCopoeia | Cat# QP004 |
| EZ-Magna RIP™ RNA-Binding Protein Immunoprecipitation Kit | Millipore | Cat# 17-701 |
| Hyperactive in-Situ ChIP kit | Vazyme | Cat# TD901-01 |
| Fluorescent in situ hybridization kit | Genepharma | Cat# F03201 |
| Dual-Luciferase Reporter Assay System | Promega | Cat# E1960 |
| **Experimental Models: Cell Lines** | | |
| Human: alveolar bone marrow-derived mesenchymal stem cells | This paper | N/A |
| Mouse: bone marrow-derived mesenchymal stem cells | This paper | N/A |
| Human: 293T cells | ATCC | CM-1009 |
| **Experimental Models: Organisms/Strains** | | |
| Mouse: miR-143/145$^{-/-}$ (B6/N-miR-143/145$^{tm1}$) | Model Animal Research Center of Nanjing | T000090 |
| **Oligonucleotides** | | |
| GMR-miRTM microRNA single-stranded mimics | Genepharma | B01001 |
| GMR-miRTM microRNA inhibitors | Genepharma | B03001 |
| miR-UPTM agomir | Genepharma | B06002 |
| miR-DownTM antagomir | Genepharma | B05002 |
| Chemically modified siRNA | Genepharma | A02004 |
| **Recombinant DNA** | | |

| | | |
|---|---|---|
| Plasmid: pEX-3 (pGCMV/MCS/Neo) vector | Genepharma | N/A |
| Plasmid: pcDNA3-MS2 system vector | GeneCopoeia | N/A |
| Plasmid: pGL3-basic luciferase reporter vector | GeneCopoeia | N/A |
| **Software and Algorithms** | | |
| ImageJ | Softonic | https://imagej.en.softonic.com/ |
| LAS V4.12 | Leica Microsystems | https://www.leica- |
| GraphPad Prism 8 | GraphPad | https://www.graphpad.com/ |
| Micro-CT NRecon v1.6 and CTAn v1.13.8.1 | Skyscan | https://www.skyscan.pt/ |
| Image-Pro Plus 7 | Media Cybernetics | http://www.mediacy.com/image |
| Adobe Illustrator | Adobe | https://www.adobe.com/cn/prod |
